# Genetic Insights Into the Role of Cathepsins in Alzheimer's Disease, Parkinson's Disease, and Amyotrophic Lateral Sclerosis: Evidence From Mendelian Randomization Study

**DOI:** 10.1002/brb3.70207

**Published:** 2024-12-31

**Authors:** Yanhong Jiang, Wenhui Fan, Yaxin Li, Hua Xue

**Affiliations:** ^1^ Department of Neurology Sichuan Taikang Hospital Chengdu Sichuan China

**Keywords:** Alzheimer's disease, amyotrophic lateral sclerosis, cathepsins, mendelian randomization, Parkinson's disease

## Abstract

**Background:**

Previous studies have confirmed the significant role of cathepsins in the development of neurodegenerative diseases. We aimed to determine whether genetically predicted 10 cathepsins may have a causal effect on Alzheimer's disease (AD), Parkinson's disease (PD), and amyotrophic lateral sclerosis (ALS).

**Methods:**

We conducted a two‐sample bidirectional Mendelian randomization (MR) study using publicly available data from genome‐wide association study (GWAS) to assess the causal associations between 10 cathepsins and three neurodegenerative diseases, including AD, PD, and ALS. We employed the following methods, including inverse variance weighting (IVW), MR‐Egger, and weighted median (WM). The results were further validated using sensitivity analysis.

**Results:**

The forward MR analysis results indicate that elevated cathepsin H levels increase the risk of AD (*p* = 0.005, odds ratio [OR] = 1.040, 95% confidence interval [CI] = 1.011–1.069), elevated cathepsin B levels decrease the risk of PD (*p* < 0.001, OR = 0.890, 95% CI = 0.831–0.954), and no significant association was found between cathepsin levels and ALS. Reverse MR analysis suggests that there is no causal association between 10 cathepsins and three neurodegenerative diseases.

**Conclusion:**

Our study provides new genetic insights into the role of cathepsin H in AD and cathepsin B in PD. However, our findings need to be further validated in a wider population, and future research should explore the potential mechanisms of cathepsins in these diseases in order to provide a basis for the development of new therapeutic strategies.

## Graphical abstract

AD, Alzheimer's disease; PD, Parkinson's disease; ALS, Amyotrophic lateral sclerosis. *In brief*: The forward Mendelian randomization analysis results indicate that elevated cathepsin H levels increase the risk of AD, elevated cathepsin B levels decrease the risk of PD. Conversely, reverse Mendelian randomization analysis suggest that no casual association between ten cathepsins and three neurodegenerative diseases.

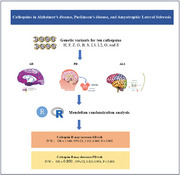

## 1 Introduction

Neurodegenerative diseases represent a group of irreversible and complex conditions characterized by progressive damage to neuronal structure and function (Nasir et al. 2024; Zhu et al. 2024). These diseases typically manifest symptoms such as cognitive decline, motor dysfunction, sensory abnormalities, and autonomic nervous system dysfunction, all of which gradually worsen over time (Nasir et al. 2024; Zhu et al. 2024). Histopathological studies reveal a slow and progressive neuronal apoptosis, with abnormal protein aggregation and inclusion bodies frequently observed in the nerve cells of affected individuals, indicating potential shared pathogenesis (Jannat et al. 2023). Alzheimer's disease (AD), Parkinson's disease (PD), and amyotrophic lateral sclerosis (ALS) are among the most prevalent neurodegenerative diseases. AD has the highest incidence among these diseases, followed by PD (Jannat et al. 2023; Liu et al. 2023). Current data suggests that over 50 million people are affected by AD, with annual medical costs projected to exceed one trillion (Zhu et al. 2024; Zhang et al. 2022). In 2019, there were approximately 8.51 million PD patients worldwide, more than double the number reported in 1990 (Kwon et al. 2021). As the aging population continues to grow, neurodegenerative diseases are expected to impose a significant medical burden. Consequently, research is increasingly focusing on the early stages of these diseases, as understanding the underlying mechanisms is essential for early prevention and treatment.

Cathepsins are the most abundant lysosomal proteases, primarily found in the acidic endo/lysosomal region (Lecaille et al. 2022; Tran and Silver 2021). They are crucial for various functions, including intracellular protein degradation, energy metabolism, and immune response. Recent research has increasingly demonstrated the significant role of cathepsins, particularly specific family members like cathepsins B and L in the pathogenesis of neurodegenerative diseases (Milanowski et al. 2022; Hook et al. 2020; Drobny et al. 2022). AD is characterized by cognitive decline and memory loss, with pathological features including senile plaques from amyloid‐β (Aβ) deposition and neurofibrillary tangles due to tau protein hyperphosphorylation (Nortley et al. 2019). Lysosomal dysfunction, particularly involving cathepsins as crucial proteolytic enzymes, is thought to be a significant factor in the progression of AD, essential for maintaining intracellular protein balance (Udayar et al. 2022). For instance, cathepsin B may contribute to the abnormal cleavage of Aβ precursor protein APP, leading to increased Aβ production (Hook et al. 2020). Clinical studies have also revealed a close link between cathepsin B levels and cognitive decline in AD patients (Y. Sun et al. 2015). Specifically, elevated cathepsin B was strongly correlated with the degree of dementia and the severity of cognitive impairment in patients, as demonstrated by the Summary Mental State Examination (MMSE) score (Y. Sun et al. 2015). Strikingly, in the brains of AD patients, cathepsin B exhibits abnormal extracellular localization, which is accompanied by the pathological characteristics of amyloid plaques in the brain (Hook et al. 2020; Cataldo and Nixon 1990). This extracellular cathepsin B contrasts with their intracellular location within lysosomes in healthy cells (Hook et al. 2020; Cataldo and Nixon 1990). Abnormal aggregation of α‐synuclein (α‐Syn) in neurons to form Lewy bodies and Lewy neurites is considered to be one of the main pathologies of PD (Olanow, Stern, and Sethi 2009). Studies have shown that increased activity of cathepsin B can effectively reduce the aggregation of α‐Syn, thereby alleviating the pathological manifestations of PD (Yusufujiang, Zeng, and Li 2024). Cathepsins play a crucial role in the development of neurodegenerative diseases, impacting proteostasis, lysosomal function, and mitochondrial dysfunction (Milanowski et al. 2022; Prieto Huarcaya et al. 2022). Understanding the pathophysiological mechanisms of cathepsins can lead to the discovery of novel biomarkers for early disease detection and the exploration of targeted treatment approaches.

As genomics continues to advance, mounting evidence is shedding light on the significant role of genetics in disease etiology (Bellenguez et al. 2022; Pihlstrøm et al. 2017). Mendelian randomization (MR) studies, a genetic method based on the genome‐wide association studies (GWAS), aim to assess the causal relationship between exposure and outcomes (Yusufujiang, Zeng, and Li 2024). Specifically, MR employs genetic variants strongly linked to particular exposure factors as instrumental variables (IVs) (Yusufujiang, Zeng, and Li 2024). These genetic variants are inherently randomly assigned at birth and are generally impervious to other confounding factors, thus theoretically mitigating or eliminating issues of confounding bias and reverse causation commonly encountered in observational studies (Yusufujiang, Zeng, and Li 2024). In this study, univariate MR methods were utilized to investigate the causal effects of various cathepsins on the susceptibility to three neurodegenerative diseases.

## 2 Materials and Methods

### 2.1 Data Sources

We obtained genetic tools for 10 cathepsin levels from the INTERVAL study, the data for which are publicly accessible at https://gwas.mrcieu.ac.uk. The INTERVAL study, a genomic bioresource, recruited 50,000 blood donors for a randomized trial on blood donation frequency across 25 centers in England. Participants completed consent forms, and the study was approved by the UK National Research Ethics Service (11/EE/0538) (B. Sun et al. 2018).

Statistics for three common neurodegenerative diseases were obtained from the analysis of several publicly available GWAS. We obtained summary statistics for AD from an updated publicly available GWAS study involving a total of 111,326 clinically diagnosed identified AD cases and 677,663 control individuals (Bellenguez et al. 2022). The PD GWAS dataset was from the International Parkinson's Disease Genomics Consortium, with 33,674 cases and 449,056 controls (Nalls et al. 2019). ALS data summary statistics were derived from a cross‐ethnic GWAS study of 29,612 ALS patients and 122,656 controls, identifying 15 risk loci (van Rheenen et al. 2021). Informed written consent was provided by all participants, and all studies were reviewed and approved by the relevant institutional ethical review boards, eliminating the need for additional ethical approvals or licenses for this MR study.

### 2.2 Study Design

**FIGURE 1:**
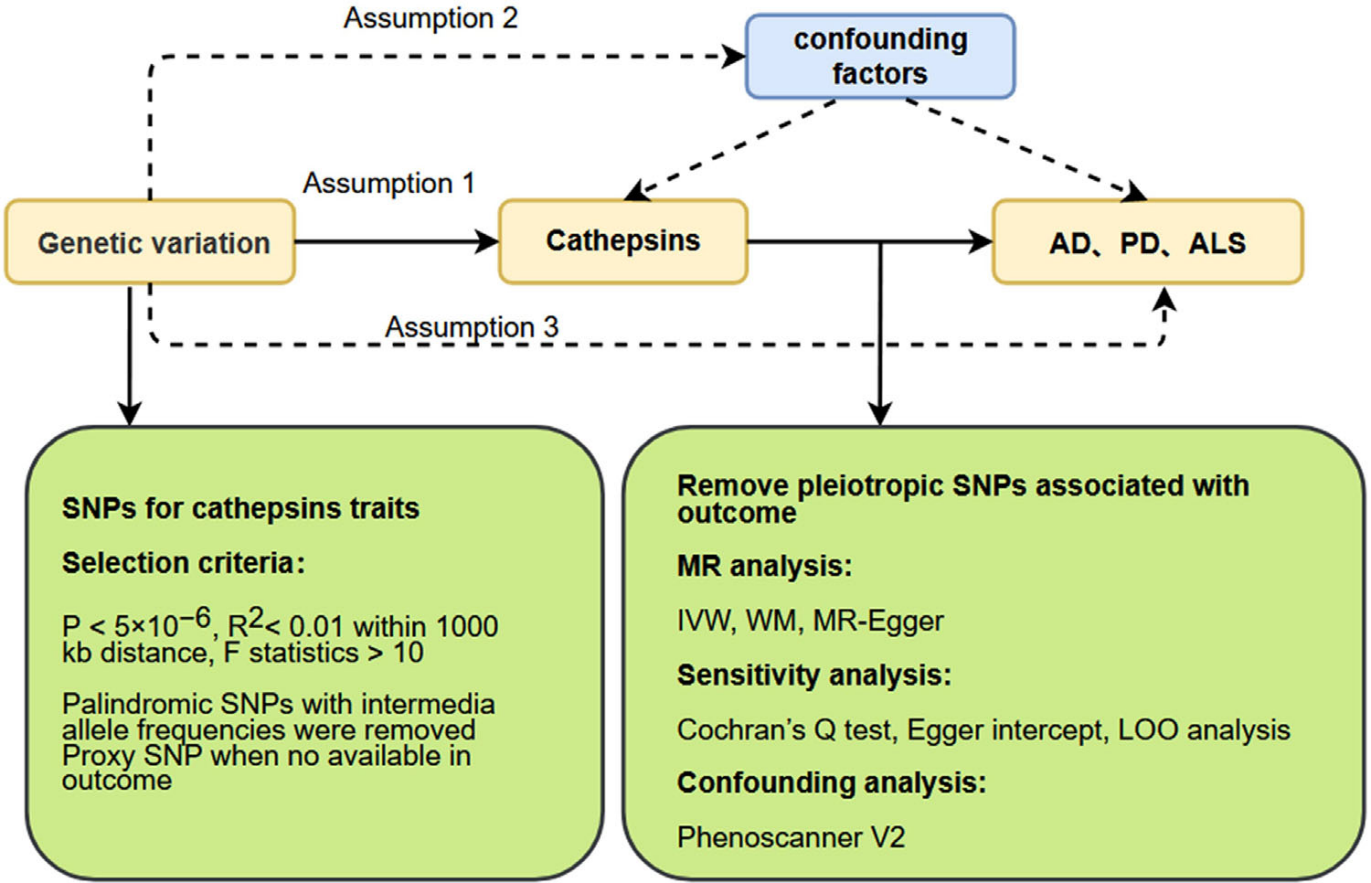
Flow chart of the study design. AD, Alzheimer's disease; ALS, Amyotrophic lateral sclerosis; IVW, inverse variance weighted; LOO, leave‐one‐out; PD, Parkinson's disease; SNP, single nucleotide polymorphism; WM, weighted median.

A bidirectional MR study was conducted to investigate the causal relationship between 10 cathepsins and three neurodegenerative diseases. Genetic variants of cathepsin and neurodegenerative diseases were used as IVs sourced from previous GWAS summary‐level data. These IVs were required to meet three critical assumptions. Assumption 1, correlation, ensured that the extracted SNPs were strongly correlated only with the exposure variable. Assumption 2, independence, confirmed that the extracted SNPs were not associated with potential confounding variables. Assumption 3, exclusivity, indicated that the identified SNPs influenced the results solely through exposure factors (Niu, Wang, and Xu 2022; T. Wang et al. 2020; J. Li, Tang, et al. 2023). Subsequently, a two‐sample MR analysis was conducted using R software (version 4.1.3) with the R package two‐sample MR (version 0.5.6). The MR analysis methods employed included inverse variance weighted, weighted median (WM), and MR Egger. Various sensitivity analyses were carried out, such as pleiotropic tests, heterogeneity tests, and leave‐one‐out (LOO) analyses. Lastly, an inverse MR approach was utilized to investigate the potential bidirectional relationship between neurodegenerative diseases and cathepsin. The study flowchart is presented in Figure 1. This MR study abided by the STROBE‐MR checklist; details can be found in Table S1.

### 2.3 IV Selection

In order to screen IVs that are strongly associated with exposure factors and establish the validity and accuracy of the causal relationship between cathepsin and neurodegenerative diseases, we followed the following steps to select IVs. First, due to the limited number of SNPs available for MR analysis, a significance threshold of *p* < 5 × 10^−6^ was set as the IVs of cathepsin based on published relevant MR studies (C. Li et al. 2022). For IVs of neurodegenerative diseases, all genetic variants significantly associated with AD (*p* < 5 × 10^−8^) were included in our MR study (C. Li et al. 2022). Second, to eliminate any existing linkage disequilibrium, we used a clustering procedure with *R* ^2^ = 0.001 and linkage distance = 10,000 kb within a 10 kb region to ensure independence of IV exposure. Third, we examined the secondary phenotype of each SNP using PhenoScanner (http://www.phenoscanner.medschl.cam.ac.uk/phenoscanner) to exclude potential pleiotropic effects of IV. We use the “harmonise_data” function to harmonize the exposure and outcome data sets, creating a new data frame that combines the exposure and outcome data. Furthermore, the strength of the selected IVs was evaluated by calculating the *F* statistic. Using the *R* ^2^ (variance in exposure explained by the selected IVs) from the Steiger test, we calculated the overall *F* statistic (Wu et al. 2023). The *F* statistic for each SNP uses the formula, $F=R^{2}\left[N-K-1\right]/K\left[1-R^{2}\right]$, where *R* ^2^, *N*, and *K* represent the estimated exposure variance explained by IVs, sample size, and number of IVs (Bowden et al. 2016). If the *F* statistic is less than 10, the SNP is considered a weak IV and excluded from the analysis to reduce the bias caused by weak IVs (Bowden et al. 2016).

### 2.4 MR Analysis

In this study, a two‐sample bidirectional MR analysis was performed. First, the researchers examined the causal relationship between 10 cathepsins (H, F, Z, G, B, S, L1, L2, O, and E) and three neurodegenerative disorders (AD, PD, and ALS), treating the histones as exposures and the neurodegenerative disorders as outcomes. Subsequently, a reverse analysis was performed using the same settings and dataset as the forward MR analysis. Three analysis methods were employed in this research: inverse variance weighting (IVW), MR‐Egger method, and the WM method, with IVW being the primary MR analysis approach. In the IVW method, the association strength of each SNP with the exposure and outcome (typically the beta coefficient derived from regression analysis) is utilized to estimate the causal effect (Yang et al. 2022). Each SNP's effect size is weighted based on the inverse of its standard error (SE), meaning that the weight is proportional to the precision of the SNP effect estimate. The weighted effects of all SNPs are then aggregated to generate a comprehensive causal effect estimate. This method assumes that all SNPs are valid IVs and that there is no pleiotropy among them, indicating that each SNP solely influences the outcome through the exposure pathway being investigated (Bouras et al. 2022). The basic idea of the WM method is that even if some SNPs may be affected by pleiotropy, as long as at least half of the SNPs are effective IVs, it can still provide an unbiased causal effect estimate. Compared with the IVW method, the WM method is more robust in the presence of pleiotropic SNPs or weak IVs because it does not rely on the assumption that all SNPs are valid IVs (Yang et al. 2022; Bouras et al. 2022). The MR‐Egger method provides consistent causal effect estimates based on the INstrument Strength Independent of Direct Effect (InSIDE) assumption, which requires that the impact of SNPs on exposure factors is independent of their pleiotropic effects on outcomes, weakening the exclusivity assumption. The MR‐Egger method also employs a modified regression analysis, wherein, in addition to incorporating the beta values representing each SNP's association with the exposure as independent variables and those reflecting the SNP‐outcome associations as dependent variables, an intercept term is integrated into the model. Should this intercept term yield a statistically significant non‐zero value, it customarily signifies the presence of pleiotropy, alluding to a scenario where genetic variants exert effects on the outcome not solely through the investigated exposure pathway but potentially via additional biological mechanisms (Bowden et al. 2015).

Heterogeneity of MR results was assessed using Cochran's *Q* test, where a *p* value below 0.05 was deemed indicative of significant heterogeneity (Bowden et al. 2016; Yang et al. 2022). Detection of pleiotropy was conducted through MR‐Egger regression and MR pleiotropy residual sum and outlier (MR‐PRESSO) (Bouras et al. 2022). Horizontal pleiotropy was identified if the *p* value was below 0.05. Subsequently, MR estimates were recalculated after excluding outlier SNPs identified by MR‐PRESSO upon finding heterogeneity or horizontal pleiotropy. Additionally, a “LOO” sensitivity analysis was performed by iteratively removing each SNP to assess the influence of specific variants on the association between exposure and outcome (Bouras et al. 2022). The remaining SNPs were then analyzed using an IVW approach.

## 3 Results

The genetic variants used to analyze the 10 cathepsins in our study were from the INTERVAL study, while the statistical data for the three neurodegenerative diseases were from various GWAS databases. For cathepsins, a genome‐wide significance threshold (*p* < 5 × 10^−6^) was set for screening, and SNPs were identified as IVs for each cathepsin in the MR analysis. The *F* statistic for each selected IV exceeded 10, indicating the absence of weak IV bias. Detailed information on SNPs is provided in the Tables S1–S3.

**FIGURE 2:**
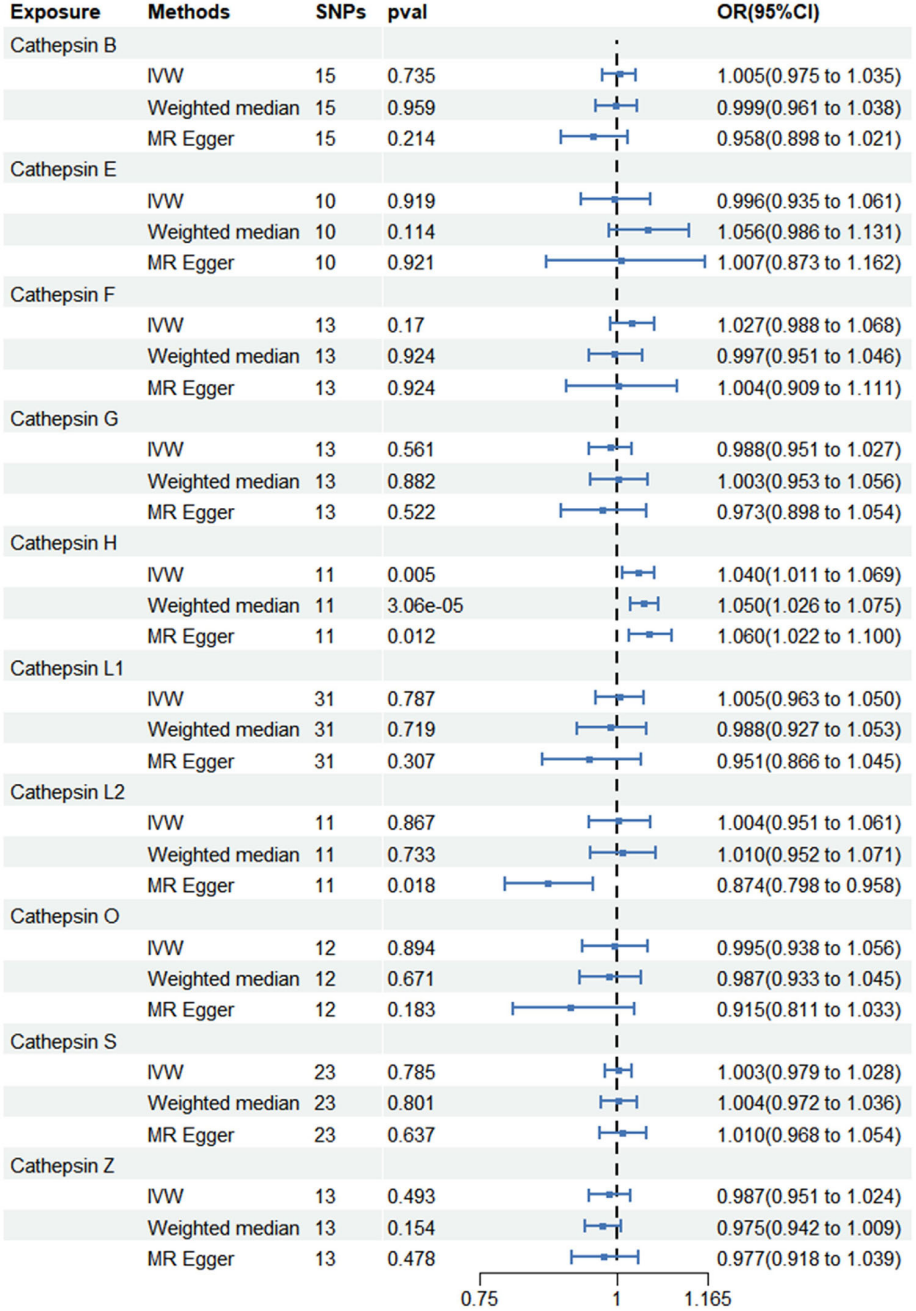
Forest plots of cathepsins on Alzheimer's disease. CI, confidence interval; IVW, inverse variance weighting; OR, odds ratio; WM, weighted median.

**FIGURE 3:**
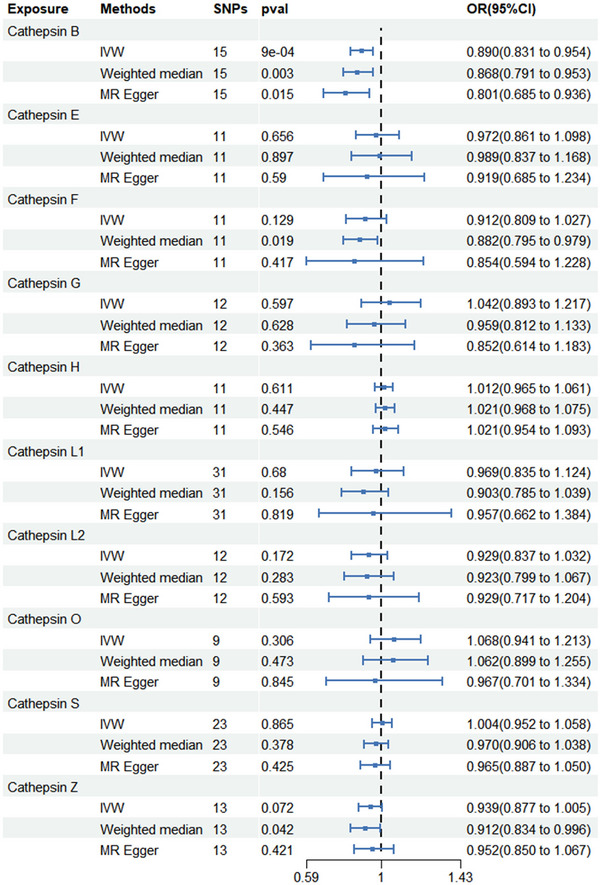
Forest plots of cathepsins on Parkinson's disease. CI, confidence interval; IVW, inverse variance weighting; OR, odds ratio; WM, weighted median;

**FIGURE 4:**
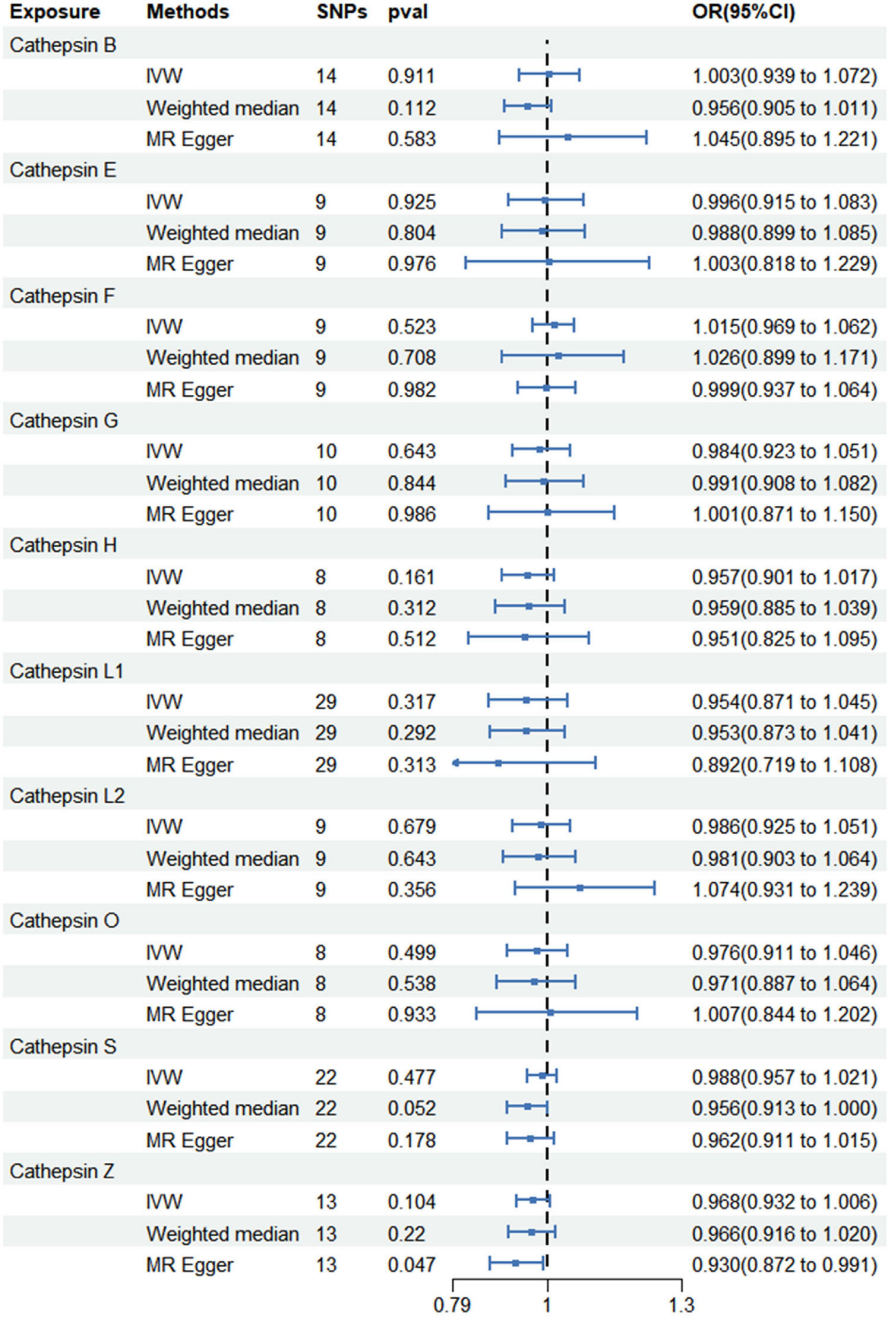
Forest plots of cathepsins on amyotrophic lateral sclerosis. CI, confidence interval; IVW, inverse variance weighting; OR, odds ratio; WM, weighted median.

Among the 10 cathepsins, IVW analysis suggested that cathepsin H increased the risk of developing AD (odds ratio [OR] = 1.040; 95% CI = 1.011–1.069; *p* = 0.005), which is consistent with the WM method (OR = 1.050; 95% CI = 1.026–1.075; *p* < 0.001) and the MR Egger (OR = 1.060; 95% CI = 1.022–1.100; *p* = 0.012) method. Cochran's *Q* test (*p* = 0.063), MR‐Egger intercept (*p* for intercept = 0.176), and MR‐PRESSO global tests (*p* = 0.2) did not yield any evidence indicating the presence of heterogeneity or horizontal pleiotropy. Scatter plots and LOO analysis further revealed that the stability of the results (Figure S1). We found no association between cathepsin B, cathepsin E, cathepsin F, cathepsin G, cathepsin L1, cathepsin L2, cathepsin O, cathepsin S, and cathepsin Z and AD risk. The results of the MR analysis estimates for the effect of cathepsins on the risk of AD are shown in Figure 2. We detected protective effects of cathepsin B on PD. Specifically, the OR of cathepsin B on PD risk was estimated to be 0.890 (95% CI = 0.831–0.954; *p* < 0.001) by using the IVW method. Similar results were observed by using WM (OR = 0.868; 95% CI = 0.791–0.953; *p* = 0.003) and MR‐Egger (OR = 0.801; 95% CI = 0.685–0.936; *p* = 0.015). The intercept of MR‐Egger (*p* for intercept = 0.162), Cochran's *Q* test (*p* = 0.453), and the global test of MR‐PRESSO (*p* = 0.26) ruled out the possibility of horizontal pleiotropy and heterogeneity. Scatter plots and funnel plots also indicated the stability of the results (Figure S2). We found no association between cathepsin H, cathepsin E, cathepsin F, cathepsin G, cathepsin L1, cathepsin L2, cathepsin O, cathepsin S, and cathepsin Z and PD risk. The results of the MR analysis estimates for the effect of cathepsins on the risk of PD are shown in Figure 3. After analysis using IVW, WM, and MR Egger methods, we found no causal link between the 10 cathepsins and ALS. The results of the three MR analysis estimates for the effect of cathepsins on the risk of ALS are displayed in Figure 4.

To address potential reverse causation, we conducted a reverse two‐sample MR analysis using three neurodegenerative diseases as exposures and 10 cathepsins as outcomes. The results of the MR analysis did not show any evidence of reverse causality (Table S4).

## 4 Discussion

The onset and progression of neurodegenerative diseases are intricate processes, with a growing body of research indicating the significant role of cathepsins (Tran and Silver 2021; Milanowski et al. 2022; Hook et al. 2020). This study systematically examined the causal relationship between 10 cathepsins and neurodegenerative diseases using genetic tools. Through two‐sample bidirectional MR analysis, potential associations between two cathepsins and two neurodegenerative diseases were observed. Specifically, higher levels of cathepsin H were linked to an increased risk of developing AD, while elevated levels of cathepsin B were associated with a potential reduction in the risk of PD. No causal relationship was found between the three neurodegenerative diseases and the 10 histones in the reverse MR analysis. These findings suggest that different cathepsins may exert varying effects on distinct types of neurodegenerative diseases.

When utilizing two‐sample bidirectional MR analysis to investigate the relationship between cathepsins and the risk of AD, it was discovered that heightened levels of cathepsin H were linked to an increased risk of AD. However, no causal relationship was identified in the reverse analysis. Cathepsin H, a member of the cysteine protease family, possesses a characteristic double‐chain structure that includes a catalytic active site and various functional regions capable of selectively cleaving peptide bonds of specific amino acid residues in the peptide chain (Y. Wang et al. 2023). Cathepsin H plays a vital role in fundamental cellular activities such as intracellular protein transport, antigen processing, and apoptosis (Ruiz‐Blázquez et al. 2021). Despite the limited number of observational studies investigating the link between cathepsin H and AD, a meta‐analysis study conducted by Y. Li, Xu, et al. (2023) based on AD‐related GWAS indicated that rs2289702 in cathepsin H might be linked to functional variations in AD. Notably, the mRNA expression levels of cathepsin H were notably elevated in AD patients and AD animal models, while human microglia with cathepsin H knockout exhibited a substantial increase in the phagocytosis of Aβ peptide (Y. Li, Xu, et al. 2023). These findings imply that cathepsin H is implicated in the genetic susceptibility of AD, shedding light on the potential genetic regulatory mechanism of cathepsin H in the pathogenesis of AD (Y. Li, Xu, et al. 2023). In addition, Cathepsin H is involved in the activation of microglia and induces the release of proinflammatory cytokines, including interleukin‐1β (IL‐1β) and tumor necrosis factor‐α (TNF‐α), which further enhance neurotoxicity and promote the occurrence of AD (Stoka et al. 2023). Previous research has demonstrated the expression, cellular localization, and activity of cathepsin B play a key role in the process of aging and aging‐related diseases (Howie, Burnett, and Crocker 1985; Bernstein et al. 1990; Nakamura et al. 1991). Immunohistochemical analysis has revealed that cathepsin B is prominently expressed in neuronal cells, particularly those located in the hippocampus (Howie, Burnett, and Crocker 1985; Bernstein et al. 1990; Nakamura et al. 1991). Further investigations by Hook et al. (2015) demonstrated a strong and selective expression of cathepsin B in hippocampal neuronal cells and the cerebral cortex of normal mice. Research indicates that cathepsin B in microglia is a key factor in the increased production of reactive oxygen species and proinflammatory mediators from mitochondria during aging, leading to cognitive dysfunction (Nakanishi 2003). Ni et al. (2019) conducted a study where they depleted the cathepsin B gene in mice and observed a significant reduction in reactive oxygen species and neuroinflammation in microglia, ultimately improving cognitive impairment associated. However, the role of cathepsin B in cognitive functions remains contentious. Embury et al. (2017) conversely suggested that overexpression of cathepsin B in hippocampal neurons could ameliorate AD‐like pathological features, such as Aβ deposition and memory deficits. Moon et al. (2016) suggested that cathepsin B may have positive effects on cognitive abilities in mice, such as enhancing hippocampal neurogenesis and spatial memory post‐exercise. Our MR analysis did not establish a causal link between cathepsin B and AD. One possible explanation for the conflicting findings on the role of cathepsin B in cognitive function is that its functions may vary across different cell types.

PD is characterized by the degeneration of dopaminergic neurons in the substantia nigra and the accumulation of Lewy bodies, primarily composed of misfolded and aggregated α‐Syn (Kwon et al. 2021). The aggregation of α‐Syn is a crucial step in the pathological process that leads to neurodegeneration in PD, with lysosomes playing a key role in the degradation of aggregated α‐Syn (Tsujimura et al. 2015; Lee et al. 2004). Mutations in lysosomal genes also represent a significant genetic risk factor for PD (Tsujimura et al. 2015; Lee et al. 2004). Cathepsin B, a lysosomal cysteine protease, is highly studied for its involvement in regulating protein degradation, autophagy, inflammatory responses, and neuronal death (Kwon et al. 2021). Previous research has linked cathepsin B to both lysosomal degradation of α‐Syn and genetic susceptibility to PD (McGlinchey and Lee 2015). Specifically, inhibition of cathepsin B impairs autophagy, reduces glucocerebrosidase (GBA1) activity, and leads to accumulation of lysosomal contents. In cell lines, reduced cathepsin B gene expression impairs degradation of preformed α‐Syn, whereas activation of cathepsin B gene enhances α‐Syn clearance (McGlinchey and Lee 2015). A GWAS study involving 6476 PD patients and 302,042 control individuals found that common variants in the gene encoding cathepsin B were associated with an increased risk of developing PD (Chang et al. 2017). Similarly, Milanowski et al. (2022) performed WES analysis on individuals in families with PD and found that the p.Gly284Val variant in the cathepsin B gene may be responsible for PD symptoms. Our forward MR analysis suggests that cathepsin B levels may be a protective factor against PD risk, consistent with previous studies.

ALS is a devastating neurodegenerative disease characterized by the progressive degeneration of upper and lower motor neurons, resulting in muscle weakness, atrophy, and ultimately death from respiratory failure (Wootz et al. 2006). The pathogenesis of ALS is highly complex and involves various factors such as genetic influences, protein misfolding, autophagy‐lysosomal dysfunction, and oxidative stress (Wootz et al. 2006; Huang et al. 2024; Kikuchi et al. 2003). Recent research has highlighted dysregulation of the lysosomal system as a crucial driver of neuronal degeneration, with cathepsins, key proteolytic enzymes within lysosomes, gaining attention for their role in ALS. Cathepsin B and L are not only present in ventral motor neurons; cathepsin B is also expressed in astrocytes. This could mean that cathepsins play a role in neuroinflammation and cell death in ALS (Tsujimura et al. 2015; Lee et al. 2004; McGlinchey and Lee 2015). Previous studies have reported that the expression of cathepsins B, L, and S is significantly increased in the spinal cord of ALS model mice (Wootz et al. 2006; Wendt, Lübbert, and Stichel 2008), whereas only cathepsin B shows up‐regulated expression in ALS patients (Offen et al. 2009). However, in our MR analysis, we did not find a genetic causal link between the 10 cathepsins and ALS. This may be attributed to the fact that MR analysis utilizes genetic variation to infer causal relationships. However, the pathogenesis of ALS likely involves a complex interplay of genetic and environmental factors, suggesting that a single genetic factor may be inadequate to establish a direct causal relationship between cathepsins and ALS. Additionally, there may be discrepancies between specific biological processes in ALS model mice and those in human disease. While increased expression of cathepsins has been observed in ALS model mice, this observation may not entirely capture the pathological processes occurring in human ALS.

This study aimed to investigate the potential causal links between various cathepsins and neurodegenerative diseases through MR analysis. The study has several strengths (Nasir et al. 2024): The assessment of causality is more precise as the associations between genetic variants and exposure factors are grounded in biological mechanisms rather than random correlations (Zhu et al. 2024). Although not strictly prospective, the random allocation of genetic variants mirrors the design of prospective studies, enhancing the credibility of the findings (Jannat et al. 2023). By mitigating potential biases from external factors, this approach provides a more reliable interpretation of the data (Liu et al. 2023). Leveraging existing large‐scale GWAS data enables a rapid and cost‐effective analysis. Nonetheless, we must also recognize the limitations of our study (Nasir et al. 2024): The database used in the study only included individuals of European ancestry. To obtain stronger evidence, it is necessary to expand the database to other ethnic groups such as Asia and Africa (Zhu et al. 2024). When screening IVs, *p* < 5 × 10^−8^ is usually considered to be the threshold indicating genome‐wide significance. In this study, we aimed to increase the number of SNPs to reduce the bias caused by the limited number of IVs, therefore; we referred to the relevant literature on MR and determined that the *p* < 5 × 10^−6^ significance threshold. Nonetheless, caution is needed when interpreting the results (Jannat et al. 2023). Epigenetic problems such as DNA methylation, RNA editing, and inactive transposons are inevitable drawbacks of MR analysis (Liu et al. 2023). The MR analysis method is a theoretical causal analysis method and needs to be further verified through animal experiments to establish a causal relationship (Zhang et al. 2022). Although MR‐Egger is valuable for detecting pleiotropy, it has low statistical power, and its validity depends on the InSIDE assumption. If the InSIDE assumption does not hold, the direct effects of genetic variants on outcomes are not independent of their effects on exposure factors, and estimates from MR‐Egger regressions will be biased (Kwon et al. 2021). The study focused on 10 cathepsins, which may limit the broad applicability of our findings.

## 5 Conclusion

In conclusion, the primary genetic evidence from this study suggests an increased risk of AD with high levels of cathepsin H and a potential protective effect of cathepsin B against PD. These findings may provide potential targets and new biomarkers for the diagnosis and treatment of neurodegenerative diseases. However, further interventional trials are needed to elucidate the underlying mechanisms.

## Author Contributions

**Yanhong Jiang**: methodology, software, writing–review and editing, writing–original draft, conceptualization. **Wenhui Fan**: writing–review and editing, software, resources, methodology. **Yaxin Li**: methodology, writing–review and editing. **Hua Xue**: software, writing–original draft, writing–review and editing, conceptualization.

## Ethics Statement

Ethical approval was not provided for this study on human participants because we used the publicly available GWAS catalog to conduct a two‐sample MR study. No additional ethical approval was required due to the re‐analysis of previously summary‐level data.

## Conflicts of Interest

The authors declare no conflicts of interest.

### Peer Review

The peer review history for this article is available at https://publons.com/publon/10.1002/brb3.70207

## Supporting information



Table S1. STROBE‐MR checklist of recommended items to address in reports of Mendelian randomization studies
Figure S1. Scatter plots of cathepsin H and AD.
Figure S2. Scatter plots of cathepsin B and PD.

Table S1. SNPs information of ten cathepsins with Alzheimer's disease
Table S2. SNPs information of ten cathepsins with amyotrophic lateral sclerosis
Table S3. SNPs information of ten cathepsins with Parkinson's disease
Table S4. Reverse MR analysis of cathepsins and neurodegenerative diseases

## Data Availability

The original contributions presented in the study are included in the article/Supplementary Material. Further inquiries can be directed to the corresponding authors.

## References

[brb370207-bib-0001] Bellenguez, C. , F. Küçükali , I. E. Jansen , et al. 2022. “New Insights Into the Genetic Etiology of Alzheimer's Disease and Related Dementias.” Nature Genetics 54, no. 4: 412–436. 10.1038/s41588-022-01024-z. 35379992 PMC9005347

[brb370207-bib-0002] Bernstein, H. G. , H. Kirschke , B. Wiederanders , D. Schmidt , and A. Rinne . 1990. “Antigenic Expression of Cathepsin B in Aged Human Brain.” Brain Research Bulletin 24, no. 4: 543–549. 10.1016/0361-9230(90)90157-u. 2357585

[brb370207-bib-0003] Bouras, E. , V. Karhunen , D. Gill , et al. 2022. “Circulating Inflammatory Cytokines and Risk of Five Cancers: A Mendelian Randomization Analysis.” BMC Medicine 20, no. 1: 3. 10.1186/s12916-021-02193-0. 35012533 PMC8750876

[brb370207-bib-0004] Bowden, J. , G. D. Smith , and S. Burgess . 2015. “Mendelian Randomization With Invalid Instruments: Effect Estimation and Bias Detection Through Egger Regression.” International Journal of Epidemiology 44, no. 2: 512–525. 10.1093/ije/dyv080. 26050253 PMC4469799

[brb370207-bib-0005] Bowden, J. , G. D. Smith , P. C. Haycock , and S. Burgess . 2016. “Consistent Estimation in Mendelian Randomization With Some Invalid Instruments Using a Weighted Median Estimator.” Genetic Epidemiology 40, no. 4: 304–314. 10.1002/gepi.21965. 27061298 PMC4849733

[brb370207-bib-0006] Cataldo, A. M. , and R. A. Nixon . 1990. “Enzymatically Active Lysosomal Proteases Are Associated With Amyloid Deposits in Alzheimer Brain.” Proceedings of the National Academy of Sciences of the United States of America 87, no. 10: 3861–3865. 10.1073/pnas.87.10.3861. 1692625 PMC54003

[brb370207-bib-0007] Chang, D. , M. A. Nalls , I. B. Hallgrímsdóttir , et al. 2017. “A Meta‐Analysis of Genome‐Wide Association Studies Identifies 17 New Parkinson's Disease Risk Loci.” Nature Genetics 49, no. 10: 1511–1516. 10.1038/ng.3955. 28892059 PMC5812477

[brb370207-bib-0008] Drobny, A. , S. Prieto Huarcaya , J. Dobert , et al. 2022. “The Role of Lysosomal Cathepsins in Neurodegeneration: Mechanistic Insights, Diagnostic Potential and Therapeutic Approaches.” Biochimica et Biophysica Acta (BBA): Molecular Cell Research 1869, no. 7: 119243. 10.1016/j.bbamcr.2022.119243. 35217144

[brb370207-bib-0009] Embury, C. M. , B. Dyavarshetty , Y. Lu , et al. 2017. “Cathepsin B Improves β‐Amyloidosis and Learning and Memory in Models of Alzheimer's Disease.” Journal of Neuroimmune Pharmacology 12, no. 2: 340–352. 10.1007/s11481-016-9721-6. 27966067 PMC5405105

[brb370207-bib-0010] Hook, G. , J. S. Jacobsen , K. Grabstein , M. Kindy , and V. Hook . 2015. “Cathepsin B Is a New Drug Target for Traumatic Brain Injury Therapeutics: Evidence for E64d as a Promising Lead Drug Candidate.” Frontiers in Neurology 6: 178. 10.3389/fneur.2015.00178. 26388830 PMC4557097

[brb370207-bib-0011] Hook, V. , M. Yoon , C. Mosier , et al. 2020. “Cathepsin B in Neurodegeneration of Alzheimer's Disease, Traumatic Brain Injury, and Related Brain Disorders.” Biochimica et Biophysica Acta (BBA): Proteins and Proteomics 1868, no. 8: 140428. 10.1016/j.bbapap.2020.140428. 32305689 PMC7261628

[brb370207-bib-0012] Howie, A. J. , D. Burnett , and J. Crocker . 1985. “The Distribution of Cathepsin B in Human Tissues.” Journal of Pathology 145, no. 4: 307–314. 10.1002/path.1711450404. 3889245

[brb370207-bib-0013] Huang, J. , Y. Yu , D. Pang , et al. 2024. “Lnc‐HIBADH‐4 Regulates Autophagy‐Lysosome Pathway in Amyotrophic Lateral Sclerosis by Targeting Cathepsin D.” Molecular Neurobiology 61: 4768–4782. 10.1007/s12035-023-03835-5. 38135852 PMC11236912

[brb370207-bib-0014] Jannat, K. , R. Balakrishnan , J. H. Han , Y. J. Yu , G. W. Kim , and D. K. Choi . 2023. “The Neuropharmacological Evaluation of Seaweed: A Potential Therapeutic Source.” Cells 12, no. 22: 00. 10.3390/cells12222652. PMC10670678 37998387

[brb370207-bib-0015] Kikuchi, H. , T. Yamada , H. Furuya , et al. 2003. “Involvement of Cathepsin B in the Motor Neuron Degeneration of Amyotrophic Lateral Sclerosis.” Acta Neuropathologica 105, no. 5: 462–468. 10.1007/s00401-002-0667-9. 12677446

[brb370207-bib-0016] Kwon, M. , M. J. Cheong , J. Leem , and T. H. Kim . 2021. “Effect of Acupuncture on Movement Function in Patients With Parkinson's Disease: Network Meta‐Analysis of Randomized Controlled Trial.” Healthcare 9, no. 11: 1502. 10.3390/healthcare9111502. 34828548 PMC8619200

[brb370207-bib-0017] Lecaille, F. , T. Chazeirat , A. Saidi , and G. Lalmanach . 2022. “Cathepsin V: Molecular Characteristics and Significance in Health and Disease.” Molecular Aspects of Medicine 88: 101086. 10.1016/j.mam.2022.101086. 35305807

[brb370207-bib-0018] Lee, H. J. , F. Khoshaghideh , S. Patel , and S. J. Lee . 2004. “Clearance of Alpha‐Synuclein Oligomeric Intermediates via the Lysosomal Degradation Pathway.” Journal of Neuroscience 24, no. 8: 1888–1896. 10.1523/jneurosci.3809-03.2004. 14985429 PMC6730405

[brb370207-bib-0019] Li, C. , J. Liu , J. Lin , and H. Shang . 2022. “Covid‐19 and Risk of Neurodegenerative Disorders: A Mendelian Randomization Study.” Translational Psychiatry 12, no. 1: 283. 10.1038/s41398-022-02052-3. 35835752 PMC9281279

[brb370207-bib-0020] Li, J. , M. Tang , X. Gao , S. Tian , and W. Liu . 2023. “Mendelian Randomization Analyses Explore the Relationship Between Cathepsins and Lung Cancer.” Communications Biology 6, no. 1: 1019. 10.1038/s42003-023-05408-7. 37805623 PMC10560205

[brb370207-bib-0021] Li, Y. , M. Xu , B. L. Xiang , et al. 2023. “Functional Genomics Identify Causal Variant Underlying the Protective CTSH Locus for Alzheimer's Disease.” Neuropsychopharmacology 48, no. 11: 1555–1566. 10.1038/s41386-023-01542-2. 36739351 PMC10516988

[brb370207-bib-0022] Liu, X. , Y. N. Ou , Y. H. Ma , L. Y. Huang , W. Zhang , and L. Tan . 2023. “Renal Function and Neurodegenerative Diseases: A Two‐Sample Mendelian Randomization Study.” Neurological Research 45, no. 5: 456–464. 10.1080/01616412.2022.2158640. 36692889

[brb370207-bib-0023] McGlinchey, R. P. , and J. C. Lee . 2015. “Cysteine Cathepsins Are Essential in Lysosomal Degradation of Α‐Synuclein.” Proceedings of the National Academy of Sciences of the United States of America 112, no. 30: 9322–9327. 10.1073/pnas.1500937112. 26170293 PMC4522768

[brb370207-bib-0024] Milanowski, L. M. , X. Hou , J. M. Bredenberg , et al. 2022. “Cathepsin B p.Gly284Val Variant in Parkinson's Disease Pathogenesis.” International Journal of Molecular Sciences 23, no. 13: 7086. 10.3390/ijms23137086. 35806091 PMC9266886

[brb370207-bib-0025] Moon, H. Y. , A. Becke , D. Berron , et al. 2016. “Running‐Induced Systemic Cathepsin B Secretion Is Associated With Memory Function.” Cell Metabolism 24, no. 2: 332–340. 10.1016/j.cmet.2016.05.025. 27345423 PMC6029441

[brb370207-bib-0026] Nakamura, Y. , M. Takeda , H. Suzuki , et al. 1991. “Abnormal Distribution of Cathepsins in the Brain of Patients With Alzheimer's Disease.” Neuroscience Letters 130, no. 2: 195–198. 10.1016/0304-3940(91)90395-a. 1795881

[brb370207-bib-0027] Nakanishi, H. 2003. “Neuronal and Microglial Cathepsins in Aging and Age‐Related Diseases.” Ageing Research Reviews 2, no. 4: 367–381. 10.1016/s1568-1637(03)00027-8. 14522241

[brb370207-bib-0028] Nalls, M. A. , C. Blauwendraat , C. L. Vallerga , et al. 2019. “Expanding Parkinson's Disease Genetics: Novel Risk Loci, Genomic Context, Causal Insights and Heritable Risk.” *BioRxiv*. 10.1101/388165.

[brb370207-bib-0029] Nasir, A. , M. U. Rehman , T. Khan , et al. 2024. “Advances in Nanotechnology‐Assisted Photodynamic Therapy for Neurological Disorders: A Comprehensive Review.” Artificial Cells, Nanomedicine, and Biotechnology 52, no. 1: 84–103. 10.1080/21691401.2024.2304814. 38235991

[brb370207-bib-0030] Ni, J. , Z. Wu , V. Stoka , et al. 2019. “Increased Expression and Altered Subcellular Distribution of Cathepsin B in Microglia Induce Cognitive Impairment Through Oxidative Stress and Inflammatory Response in Mice.” Aging Cell 18, no. 1: e12856. 10.1111/acel.12856. 30575263 PMC6351837

[brb370207-bib-0031] Niu, P. P. , X. Wang , and Y. M. Xu . 2022. “Higher Circulating Vitamin D Levels Are Associated With Decreased Migraine Risk: A Mendelian Randomization Study.” Frontiers in Nutrition 9: 907789. 10.3389/fnut.2022.907789. 36159496 PMC9505695

[brb370207-bib-0032] Nortley, R. , N. Korte , P. Izquierdo , et al. 2019. “Amyloid β Oligomers Constrict Human Capillaries in Alzheimer's Disease via Signaling to Pericytes.” Science 365, no. 6450: eaav9518. 10.1126/science.aav9518. 31221773 PMC6658218

[brb370207-bib-0033] Offen, D. , Y. Barhum , E. Melamed , N. Embacher , C. Schindler , and G. Ransmayr . 2009. “Spinal Cord mRNA Profile in Patients With ALS: Comparison With Transgenic Mice Expressing the Human SOD‐1 Mutant.” Journal of Molecular Neuroscience 38, no. 2: 85–93. 10.1007/s12031-007-9004-z. 18651250

[brb370207-bib-0034] Olanow, C. W. , M. B. Stern , and K. Sethi . 2009. “The Scientific and Clinical Basis for the Treatment of Parkinson Disease (2009).” Neurology 72, no. 21_S4: S1–136. 10.1212/WNL.0b013e3181a1d44c. 19470958

[brb370207-bib-0035] Pihlstrøm, L. , S. Wiethoff , and H. Houlden . 2017. “Genetics of Neurodegenerative Diseases: An Overview.” In Handbook of Clinical Neurology, edited by G. G. Kovacs and I. Alafuzoff , 309–323. Elsevier. 10.1016/b978-0-12-802395-2.00022-5. 28987179

[brb370207-bib-0036] Prieto Huarcaya, S. , A. Drobny , A. R. A. Marques , et al. 2022. “Recombinant Pro‐CTSD (Cathepsin D) Enhances SNCA/α‐Synuclein Degradation in α‐Synucleinopathy Models.” Autophagy 18, no. 5: 1127–1151. 10.1080/15548627.2022.2045534. 35287553 PMC9196656

[brb370207-bib-0037] Ruiz‐Blázquez, P. , V. Pistorio , M. Fernández‐Fernández , and A. Moles . 2021. “The Multifaceted Role of Cathepsins in Liver Disease.” Journal of Hepatology 75, no. 5: 1192–1202. 10.1016/j.jhep.2021.06.031. 34242696

[brb370207-bib-0038] Stoka, V. , O. Vasiljeva , H. Nakanishi , and V. Turk . 2023. “The Role of Cysteine Protease Cathepsins B, H, C, and X/Z in Neurodegenerative Diseases and Cancer.” International Journal of Molecular Sciences 24, no. 21: 15613. 10.3390/ijms242115613. 37958596 PMC10650516

[brb370207-bib-0039] Sun, B. B. , J. C. Maranville , J. E. Peters , et al. 2018. “Genomic Atlas of the Human Plasma Proteome.” Nature 558, no. 7708: 73–79. 10.1038/s41586-018-0175-2. 29875488 PMC6697541

[brb370207-bib-0040] Sun, Y. , X. Rong , W. Lu , et al. 2015. “Translational Study of Alzheimer's Disease (AD) Biomarkers From Brain Tissues in AβPP/PS1 Mice and Serum of AD Patients.” Journal of Alzheimer's Disease 45, no. 1: 269–282. 10.3233/jad-142805. 25502766

[brb370207-bib-0041] Tran, A. P. , and J. Silver . 2021. “Cathepsins in Neuronal Plasticity.” Neural Regeneration Research 16, no. 1: 26–35. 10.4103/1673-5374.286948. 32788444 PMC7818855

[brb370207-bib-0042] Tsujimura, A. , K. Taguchi , Y. Watanabe , et al. 2015. “Lysosomal Enzyme Cathepsin B Enhances the Aggregate Forming Activity of Exogenous α‐Synuclein Fibrils.” Neurobiology of Disease 73: 244–253. 10.1016/j.nbd.2014.10.011. 25466281

[brb370207-bib-0043] Udayar, V. , Y. Chen , E. Sidransky , and R. Jagasia . 2022. “Lysosomal Dysfunction in Neurodegeneration: Emerging Concepts and Methods.” Trends in Neuroscience 45, no. 3: 184–199. 10.1016/j.tins.2021.12.004. PMC8854344 35034773

[brb370207-bib-0044] van Rheenen, W. , R. A. A. van der Spek , M. K. Bakker , et al. 2021. “Common and Rare Variant Association Analyses in Amyotrophic Lateral Sclerosis Identify 15 Risk Loci With Distinct Genetic Architectures and Neuron‐Specific Biology.” Nature Genetics 53, no. 12: 1636–1648. 10.1038/s41588-021-00973-1. 34873335 PMC8648564

[brb370207-bib-0045] Wang, T. , Q. B. Ni , K. Wang , Z. Han , and B. L. Sun . 2020. “Stroke and Alzheimer's Disease: A Mendelian Randomization Study.” Frontiers in Genetics 11: 581. 10.3389/fgene.2020.00581. 32760421 PMC7371994

[brb370207-bib-0046] Wang, Y. , J. Zhao , Y. Gu , et al. 2023. “Cathepsin H: Molecular Characteristics and Clues to Function and Mechanism.” Biochemical Pharmacology 212: 115585. 10.1016/j.bcp.2023.115585. 37148981

[brb370207-bib-0047] Wendt, W. , H. Lübbert , and C. C. Stichel . 2008. “Upregulation of Cathepsin S in the Aging and Pathological Nervous System of Mice.” Brain Research 1232: 7–20. 10.1016/j.brainres.2008.07.067. 18694734

[brb370207-bib-0048] Wootz, H. , E. Weber , L. Korhonen , and D. Lindholm . 2006. “Altered Distribution and Levels of CathepsinD and Cystatins in Amyotrophic Lateral Sclerosis Transgenic Mice: Possible Roles in Motor Neuron Survival.” Neuroscience 143, no. 2: 419–430. 10.1016/j.neuroscience.2006.07.048. 16973300

[brb370207-bib-0049] Wu, Q. , S. Liu , X. Huang , et al. 2023. “Bidirectional Mendelian Randomization Study of Psychiatric Disorders and Parkinson's Disease.” Frontiers in Aging Neuroscience 15: 1120615. 10.3389/fnagi.2023.1120615. 36998320 PMC10045982

[brb370207-bib-0050] Yang, F. , T. Hu , K. He , J. Ying , and H. Cui . 2022. “Multiple Sclerosis and the Risk of Cardiovascular Diseases: A Mendelian Randomization Study.” Frontiers in Immunology 13: 861885. 10.3389/fimmu.2022.861885. 35371017 PMC8964627

[brb370207-bib-0051] Yusufujiang, A. , S. Zeng , and H. Li . 2024. “Cathepsins and Parkinson's Disease: Insights From Mendelian Randomization Analyses.” Frontiers in Aging Neuroscience 16: 1380483. 10.3389/fnagi.2024.1380483. 38903897 PMC11188310

[brb370207-bib-0052] Zhang, D. , S. Chen , S. Xu , et al. 2022. “The Clinical Correlation Between Alzheimer's Disease and Epilepsy.” Frontiers in Neurology 13: 922535. 10.3389/fneur.2022.922535. 35937069 PMC9352925

[brb370207-bib-0053] Zhu, F. , S. Yin , T. Ma , et al. 2024. “An Overview of Systematic Reviews of Acupuncture for Neurodegenerative Disease.” Asian Journal of Psychiatry 91: 103882. 10.1016/j.ajp.2023.103882. 38150809

