# Supplementary material for: Genetic Insights Into the Role of Cathepsins in Alzheimer's Disease, Parkinson's Disease, and Amyotrophic Lateral Sclerosis: Evidence From Mendelian Randomization Study
Source: Brain Behav. 2024 Dec 31;15(1):e70207. doi: 10.1002/brb3.70207 (PMC11688054; doi:10.1002/brb3.70207)
Supplement: Supplementary file 1 — Table S1. STROBE‐MR checklist of recommended items to address in reports of Mendelian randomization studies Figure S1. Scatter plots of cathepsin H and AD. Figure S2. Scatter plots of cathepsin B and PD. [file BRB3-15-e70207-s001.docx]

**Table S1.** STROBE-MR checklist of recommended items to address in reports of Mendelian randomization studies

| **Item No.** | **Section** | **Checklist item** | **Page No.** | **Relevant text from manuscript** |
| --- | --- | --- | --- | --- |
| 1 | **TITLE and**  **ABSTRACT** | Indicate Mendelian randomization (MR) as the study’s design in the title and/or the abstract if that is a main  purpose of the study | 1-2 | Genetic insights into the role of cathepsins in neurodegenerative diseases: evidence from Mendelian randomization study |
|  | **INTRODUCTION** |  |  |  |
| 2 | **Background** | Explain the scientific background and rationale for the reported study. What is the exposure? Is a potential causal relationship between exposure and outcome plausible? Justify why MR is a helpful method to address the study  question | 2-4 | Mendelian randomization (MR) analysis is a research method that utilizes genetic variation as instrumental variables (IVs) to identify and detect the causal effects of exposure on outcomes. |
| 3 | **Objectives** | State specific objectives clearly, including pre-specified causal hypotheses (if any). State that MR is a method  that, under specific assumptions, intends to estimate causal effects | 4 | In this study, univariate Mendelian randomization methods were utilized to investigate the causal effects of various cathepsins on the susceptibility to three neurodegenerative diseases. |
|  | **METHODS** |  |  |  |
| 4 | **Study design and data**  **sources** | Present key elements of the study design early in the article. Consider including a table listing sources of data  for all phases of the study. For each data source contributing to the analysis, describe the following: |  |  |
|  | a) | Setting: Describe the study design and the underlying population, if possible. Describe the setting, locations, and  relevant dates, including periods of recruitment, exposure, follow-up, and data collection, when available. | 4-7 | A bidirectional Mendelian randomization study was conducted to investigate the causal relationship between ten cathepsins and three neurodegenerative diseases. Genetic variants of cathepsin and neurodegenerative diseases were used as instrumental variables (IVs) sourced from previous GWAS summary-level data. |
|  | b) | Participants: Give the eligibility criteria, and the sources and methods of selection of participants. Report the  sample size, and whether any power or sample size calculations were carried out prior to the main analysis | 4-7 | We obtained summary statistics for Alzheimer's disease (AD) from an updated publicly available GWAS study involving a total of 111,326 clinically diagnosed identified AD cases and 677,663 control individuals . The Parkinson's Disease GWAS dataset was from the International Parkinson's Disease Genomics Consortium, with 33,674 cases and 449,056 controls . Amyotrophic lateral sclerosis (ALS) data summary statistics were derived from a cross-ethnic GWAS study of 29,612 ALS patients and 122,656 controls, identifying 15 risk loci . |
|  | c) | Describe measurement, quality control and selection of genetic variants | 4-7 | These IVs were required to meet three critical assumptions. Assumption 1, correlation, ensured that the extracted SNPs were strongly correlated only with the exposure variable. Assumption 2, independence, confirmed that the extracted SNPs were not associated with potential confounding variables. Assumption 3, exclusivity, indicated that the identified SNPs influenced the results solely through exposure factors |
|  | d) | For each exposure, outcome, and other relevant variables, describe methods of assessment and diagnostic criteria  for diseases | 4-7 | The INTERVAL study, a genomic bioresource, recruited 50,000 blood donors for a randomized trial on blood donation frequency across 25 centers in England. Participants completed consent forms, and the study was approved by the UK National Research Ethics Service (11/EE/0538) |
|  | e) | Provide details of ethics committee approval and participant informed consent, if relevant | 4-7 | All included original studies obtained ethical review approval and informed consent from the participants. |
| 5 | **Assumptions** | Explicitly state the three core IV assumptions for the main analysis (relevance, independence and exclusion  restriction) as well assumptions for any additional or sensitivity analysis | 5 | MR analysis must satisfy three assumptions. |
| 6 | **Statistical methods:**  **main analysis** | Describe statistical methods and statistics used |  |  |
|  | a) | Describe how quantitative variables were handled in the analyses (i.e., scale, units, model) | 5-7 | We performed a two-sample Mendelian randomization analysis using three MR methods, including inverse variance weighted (IVW), MR-Egger regression, weighted median. |
|  | b) | Describe how genetic variants were handled in the analyses and, if applicable, how their weights were selected | 6 | For IVs of inflammatory cytokines, we used a more relaxed threshold to obtain more single nucleotide polymorphisms (SNPs), setting the parameter to P < 5×10^-6^, which has been used in many other MR studies. |
|  | c) | Describe the MR estimator (e.g. two-stage least squares, Wald ratio) and related statistics. Detail the included  covariates and, in case of two-sample MR, whether the same covariate set was used for adjustment in the two  samples | 4-5 | The causal association could be evaluated……measure heterogeneity among the estimates from each SNP. |
|  | d) | Explain how missing data were addressed | 6 | If the SNP as IV contain missing data in the exposure or outcome  summary, it would be omitted. |
|  | e) | If applicable, indicate how multiple testing was addressed | 7 | Heterogeneity of MR results was assessed using Cochran’s Q test, where a P value below 0.05 was deemed indicative of significant heterogeneity |
| 7 | **Assessment of**  **assumptions** | Describe any methods or prior knowledge used to assess the assumptions or justify their validity | 6 | To avoid bias from weak IVs, we performed strength calculations on screened IVs using variance (R2) and F-statistics. |
| 8 | **Sensitivity analyses**  **and additional analyses** | Describe any sensitivity analyses or additional analyses performed (e.g. comparison of effect estimates from different approaches, independent replication, bias analytic techniques, validation of instruments, simulations) | 8 | We performed a series of sensitivity tests on MR results, including heterogeneity tests, multiple validity tests, and leave-one-out sensitivity tests. |
| 9 | **Software and pre-**  **registration** |  |  |  |
|  | a) | Name statistical software and package(s), including version and settings used | 8 | All analyses were performed using R, version 4.0.2. |
|  | b) | State whether the study protocol and details were pre-registered (as well as when and where) | 8 | The study was not pre-registered on any platform. |
|  | **RESULTS** |  |  |  |
| 10 | **Descriptive data** |  |  |  |
|  | a) | Report the numbers of individuals at each stage of included studies and reasons for exclusion. Consider use of a flow diagram | 7 | Figure 1 |
|  | b) | Report summary statistics for phenotypic exposure(s), outcome(s), and other relevant variables (e.g. means, SDs, proportions) | 7 | Supplementary material |
|  | c) | If the data sources include meta-analyses of previous studies, provide the assessments of heterogeneity across  these studies | Not  available | Not provided in original research |
|  | d) | For two-sample MR:   1. Provide justification of the similarity of the genetic variant-exposure associations between the exposure and outcome samples 2. Provide information on the number of individuals who overlap between the exposure and outcome studies | 7 | There would be no overlap in population selection between exposure group and outcome group. |
| 11 | **Main results** |  |  |  |
|  | a) | Report the associations between genetic variant and exposure, and between genetic variant and outcome, preferably on an interpretable scale | 7-8 | Supplementary material |
|  | b) | Report MR estimates of the relationship between exposure and outcome, and the measures of uncertainty from the MR analysis, on an interpretable scale, such as odds ratio or relative risk per SD difference | 7-8 | Among the 10 cathepsins, IVW analysis suggested that cathepsin H increased the risk of developing AD (OR, 1.040; 95% CI, 1.011-1.069; P = 0.005), which is consistent with the WM method (OR, 1.050; 95% CI, 1.026-1.075; P < 0.001) and the MR Egger (OR, 1.060; 95% CI, 1.022-1.100; P = 0.012) method. |
|  | c) | If relevant, consider translating estimates of relative risk into absolute risk for a meaningful time period | No  relavant |  |
|  | d) | Consider plots to visualize results (e.g. forest plot, scatterplot of associations between genetic variants and  outcome versus between genetic variants and exposure) | Figure 2-4 |  |
| 12 | **Assessment of**  **assumptions** |  |  |  |
|  | a) | Report the assessment of the validity of the assumptions | Table | Supplementary Tables |
|  | b) | Report any additional statistics (e.g., assessments of heterogeneity across genetic variants, such as *I^2^*, Q statistic or E-value) | Table | Supplementary Tables |
| 13 | **Sensitivity analyses**  **and additional analyses** |  |  |  |
|  | a) | Report any sensitivity analyses to assess the robustness of the main results to violations of the assumptions | 7-8 | Cochran’ s Q test (P = 0.063), MR-Egger intercept (P for intercept = 0.176), and MR-PRESSO global tests (P = 0.2) did not yield any evidence indicating the presence of heterogeneity or horizontal pleiotropy. Scatter plots and LOO analysis further revealed that the stability of the results (Supplementary Figure 1) |
|  | b) | Report results from other sensitivity analyses or additional analyses | 7-8 | The intercept of MR-Egger (P for intercept = 0.162), Cochran’ s Q test (P = 0.453), global test of MR-PRESSO (P = 0.26) ruled out the possibility of horizontal pleiotropy and heterogeneity. Scatter plots and funnel plots also indicated the stability of the results |
|  | c) | Report any assessment of direction of causal relationship (e.g., bidirectional MR) | 7-8 | To address potential reverse causation, we conducted a reverse Two-SampleMR analysis using three neurodegenerative diseases as exposures and ten cathepsins as outcomes. The results of the MR analysis did not show any evidence of reverse causality (Table S4). |
|  | d) | When relevant, report and compare with estimates from non-MR analyses | No |  |
|  | e) | Consider additional plots to visualize results (e.g., leave-one-out analyses) | Figure  S1-S2 | Figure S1-S2 |
|  | **DISCUSSION** |  |  |  |
| 14 | **Key results** | Summarize key results with reference to study objectives | 8-9 | Specifically, higher levels of cathepsin H were linked to an increased risk of developing AD, while elevated levels of cathepsin B were associated with a potential reduction in the risk of PD. No causal relationship was found between the three neurodegenerative diseases and the ten histones in the reverse MR analysis. These findings suggest that different cathepsins may exert varying effects on distinct types of neurodegenerative diseases. |
| 15 | **Limitations** | Discuss limitations of the study, taking into account the validity of the IV assumptions, other sources of potential bias, and imprecision. Discuss both direction and magnitude of any potential bias and any efforts to address them | 10-11 | However, it is important to acknowledge the limitations of our study. |
| 16 | **Interpretation** |  |  |  |
|  | a) | Meaning: Give a cautious overall interpretation of results in the context of their limitations and in comparison with other studies | 8-11 | The study has several strengths: (1) The assessment of causality is more precise as the associations between genetic variants and exposure factors are grounded in biological mechanisms rather than random correlations. (2) Although not strictly prospective, the random allocation of genetic variants mirrors the design of prospective studies, enhancing the credibility of the findings. (3) By mitigating potential biases from external factors, this approach provides a more reliable interpretation of the data. (4) Leveraging existing large-scale genome-wide association study (GWAS) data enables a rapid and cost-effective analysis. |
|  | b) | Mechanism: Discuss underlying biological mechanisms that could drive a potential causal relationship between the investigated exposure and the outcome, and whether the gene-environment equivalence assumption is reasonable. Use causal language carefully, clarifying that IV estimates may provide causal effects only under  certain assumptions | 8-11 | Research indicates that cathepsin B in microglia is a key factor in the increased production of reactive oxygen species and pro-inflammatory mediators from mitochondria during aging, leading to cognitive dysfunction (36). Ni et al. conducted a study where they depleted the cathepsin B gene in mice and observed a significant reduction in reactive oxygen species and neuroinflammation in microglia, ultimately improving cognitive impairment associated (37). However, the role of cathepsin B in cognitive functions remains contentious. Embury et al., conversely, suggested that overexpression of cathepsin B in hippocampal neurons could ameliorate AD-like pathological features, such as Aβ deposition and memory deficits |
|  | c) | Clinical relevance: Discuss whether the results have clinical or public policy relevance, and to what extent they  inform effect sizes of possible interventions | 8-11 | Stem cells have emerged as a potential therapy for a range of neurological injuries, and their use in AD has been extensively studied in recent years. |
| 17 | **Generalizability** | Discuss the generalizability of the study results (a) to other populations, (b) across other exposure periods/timings, and (c) across other levels of exposure | 8-11 | it is important to exercise caution in generalizing our findings to individuals of other ancestries, as our study subjects were all of European descent. |
|  | **OTHER**  **INFORMATION** |  |  |  |
| 18 | **Funding** | Describe sources of funding and the role of funders in the present study and, if applicable, sources of funding for the databases and original study or studies on which the present study is based | 11 | This research did not receive any specific grant from funding agencies in the public, commercial, or not-for-profit sectors. |
| 19 | **Data and data sharing** | Provide the data used to perform all analyses or report where and how the data can be accessed, and reference these sources in the article. Provide the statistical code needed to reproduce the results in the article, or report  whether the code is publicly accessible and if so, where | 11 | The original contributions presented in the study are included in the article/Supplementary Material. Further inquiries can be directed to the corresponding authors. |
| 20 | **Conflicts of Interest** | All authors should declare all potential conflicts of interest | 11 | The authors declare that the research was conducted in the absence of any commercial or financial relationships that could be construed as a potential conflict of interest. |

| 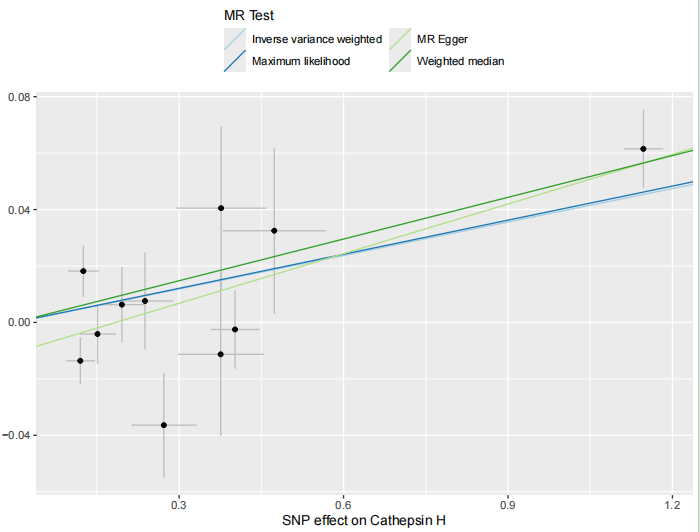 | 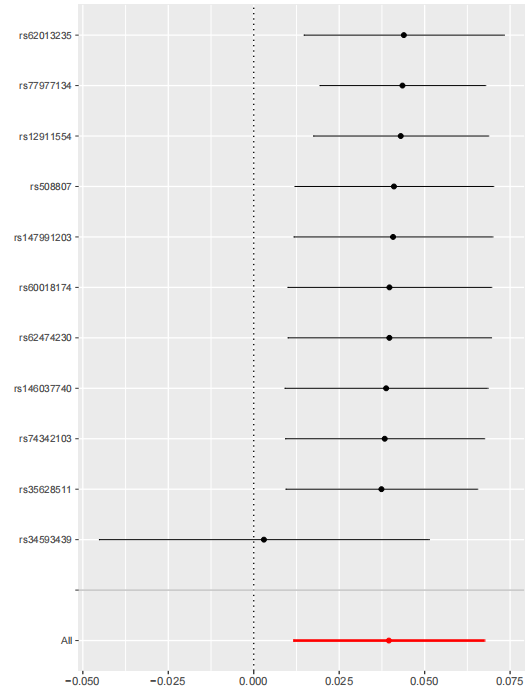 |
| --- | --- |
| Figure S1. Scatter plots of cathepsin H and AD. | LOO analysis between cathepsin H and AD. |

| 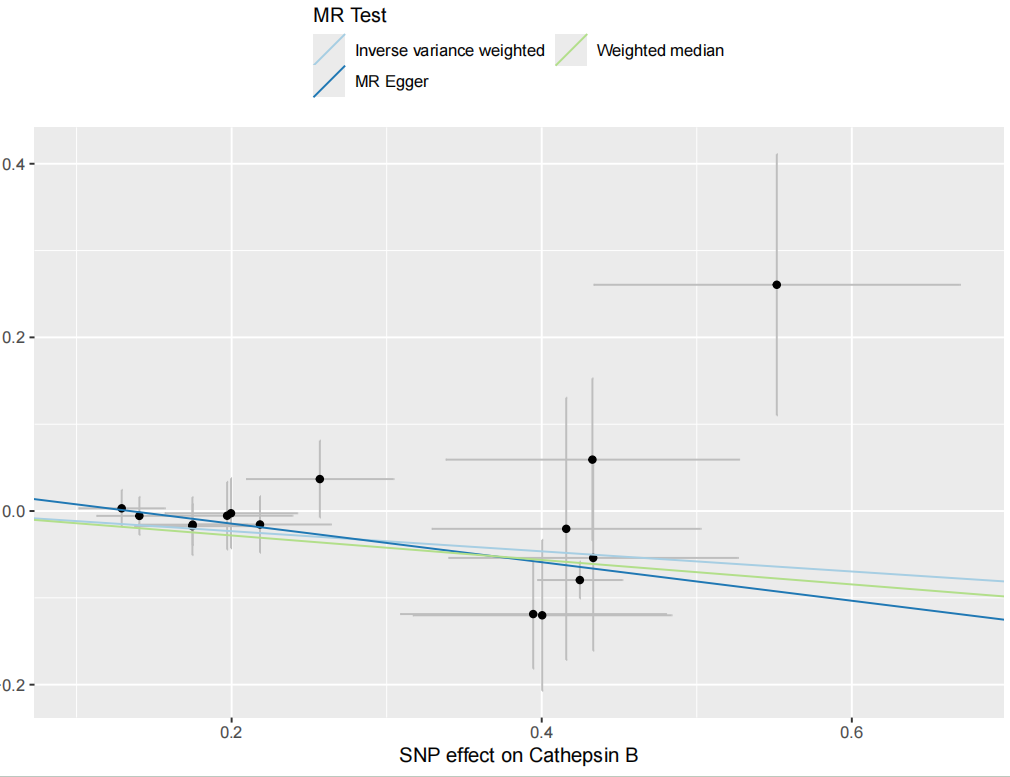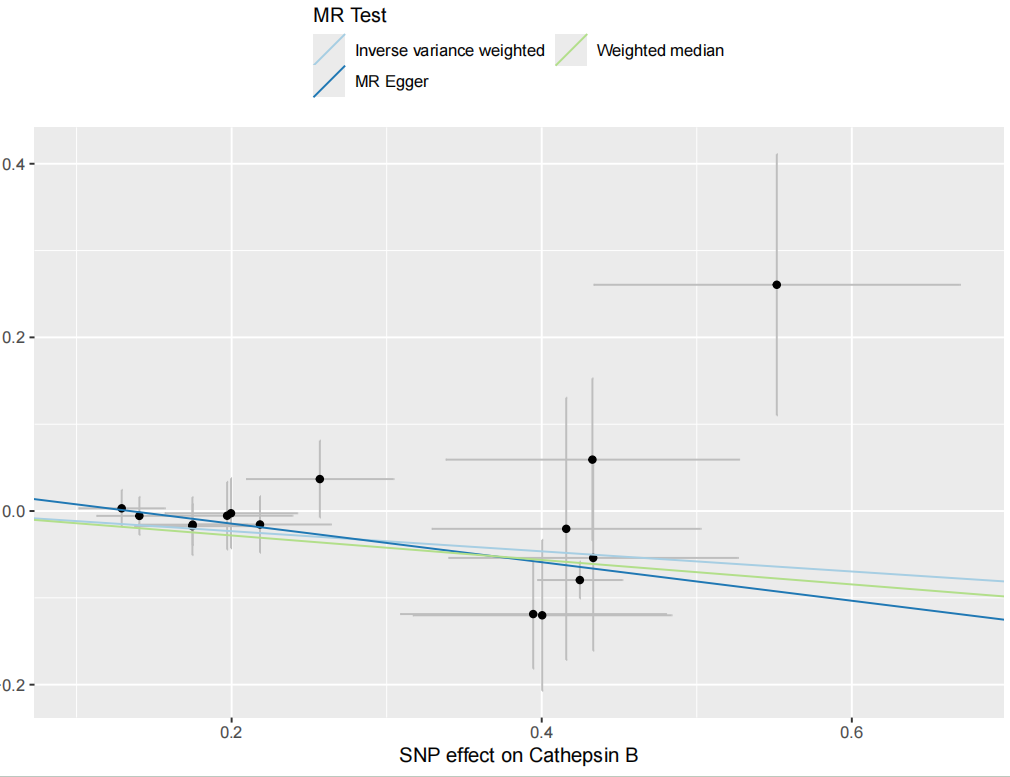 | 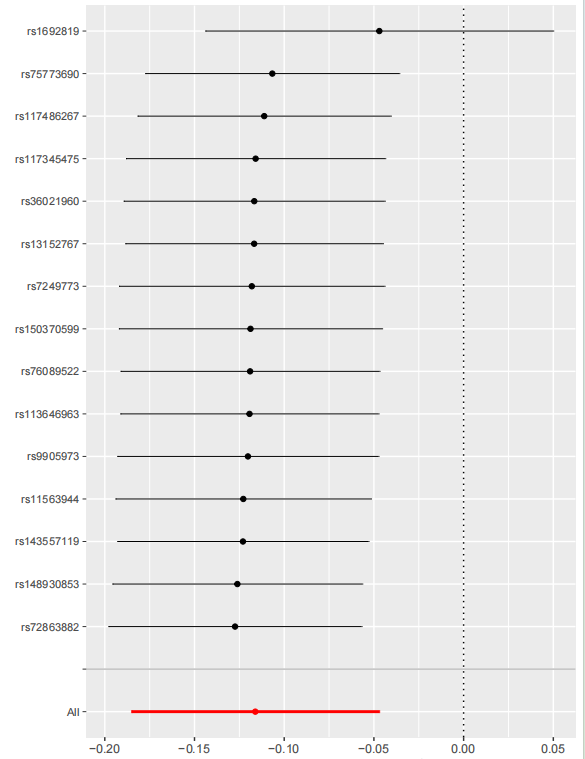 |
| --- | --- |
| Figure S2. Scatter plots of cathepsin B and PD. | LOO analysis between cathepsin B and PD. |
