# Supplementary material for: Genetic Insights Into the Role of Cathepsins in Alzheimer's Disease, Parkinson's Disease, and Amyotrophic Lateral Sclerosis: Evidence From Mendelian Randomization Study
Source: Brain Behav. 2024 Dec 31;15(1):e70207. doi: 10.1002/brb3.70207 (PMC11688054; doi:10.1002/brb3.70207)
Supplement: Supplementary file 2 — Table S1. SNPs information of ten cathepsins with Alzheimer's disease Table S2. SNPs information of ten cathepsins with amyotrophic lateral sclerosis Table S3. SNPs information of ten cathepsins with Parkinson's disease Table S4. Reverse MR analysis of cathepsins and neurodegenerative diseases [file BRB3-15-e70207-s002.docx]

| **Table S1. SNPs information of ten cathepsins with Alzheimer's disease** | | | | | | | | | | | | |
| --- | --- | --- | --- | --- | --- | --- | --- | --- | --- | --- | --- | --- |
| **Ten cathepsins** | | | | | | | | | | | **Alzheimer's disease** | |
| SNP | effect_allele | other_allele | beta | eaf | se | pval | exposure | sd | R2 | F | se | pval |
| rs113646963 | T | C | 0.1996 | 0.09708 | 0.0427 | 2.95E-06 | Cathepsin B | 8.635435004 | 0.000534001 | 21.85069846 | 0.0141 | 0.399 |
| rs11563944 | G | A | 0.1291 | 0.26799 | 0.0278 | 3.55E-06 | Cathepsin B | 5.622133328 | 0.000527039 | 21.56566689 | 0.0092 | 0.0912998 |
| rs117345475 | G | A | -0.4332 | 0.02062 | 0.0933 | 3.47E-06 | Cathepsin B | 18.8685266 | 0.000526858 | 21.55825519 | 0.0334 | 0.2451 |
| rs117486267 | C | T | -0.4003 | 0.02486 | 0.0834 | 1.58E-06 | Cathepsin B | 16.86639998 | 0.000562993 | 23.0376979 | 0.0295 | 0.1184 |
| rs143557119 | A | G | 0.4327 | 0.02199 | 0.0946 | 4.79E-06 | Cathepsin B | 19.13143212 | 0.000511303 | 20.92143732 | 0.0336 | 0.8859 |
| rs148930853 | C | T | 0.5516 | 0.01211 | 0.1181 | 3.02E-06 | Cathepsin B | 23.88395489 | 0.000533121 | 21.81467363 | 0.042 | 0.4101 |
| rs150370599 | T | C | 0.2183 | 0.08341 | 0.0457 | 1.78E-06 | Cathepsin B | 9.24214004 | 0.000557624 | 22.81786841 | 0.0153 | 0.4236 |
| rs150811995 | C | A | 0.4603 | 0.01878 | 0.1004 | 4.57E-06 | Cathepsin B | 20.30439519 | 0.000513689 | 21.01911974 | 0.0442 | 0.5547 |
| rs1692819 | A | G | 0.4246 | 0.29182 | 0.0274 | 5.25E-54 | Cathepsin B | 5.541239323 | 0.005837472 | 240.1368746 | 0.0093 | 0.8024 |
| rs36021960 | A | G | 0.1747 | 0.12637 | 0.0374 | 3.02E-06 | Cathepsin B | 7.563589442 | 0.000533236 | 21.81939003 | 0.0121 | 0.8074 |
| rs7249773 | A | G | 0.1749 | 0.15121 | 0.0351 | 6.03E-07 | Cathepsin B | 7.098448914 | 0.000606751 | 24.82935203 | 0.012 | 0.1277 |
| rs72863882 | A | G | -0.2569 | 0.08353 | 0.0475 | 6.46E-08 | Cathepsin B | 9.606163061 | 0.000714725 | 29.25101828 | 0.0159 | 0.0999793 |
| rs75773690 | A | G | -0.3945 | 0.02192 | 0.0857 | 4.17E-06 | Cathepsin B | 17.33154051 | 0.000517864 | 21.19006902 | 0.0278 | 0.7981 |
| rs76089522 | A | G | -0.1971 | 0.10475 | 0.0421 | 2.88E-06 | Cathepsin B | 8.514093997 | 0.000535655 | 21.91841053 | 0.0138 | 0.2521 |
| rs9905973 | A | G | -0.1405 | 0.271 | 0.0276 | 3.55E-07 | Cathepsin B | 5.581686326 | 0.000633239 | 25.91400179 | 0.0091 | 0.7899 |
| rs10401027 | A | G | -0.1225 | 0.45246 | 0.0259 | 2.24E-06 | Cathepsin E | 5.237886806 | 0.000546693 | 22.37034332 | 0.0085 | 0.0794401 |
| rs10900907 | A | G | 0.1188 | 0.41707 | 0.0255 | 3.24E-06 | Cathepsin E | 5.156992801 | 0.000530433 | 21.70463668 | 0.0085 | 0.271 |
| rs112918835 | T | C | 0.7062 | 0.00706 | 0.1526 | 3.72E-06 | Cathepsin E | 30.8610628 | 0.000523393 | 21.41639455 | 0.0436 | 0.1499 |
| rs13089837 | C | T | -0.1152 | 0.49848 | 0.025 | 4.17E-06 | Cathepsin E | 5.055875295 | 0.000518929 | 21.233664 | 0.0083 | 0.0419295 |
| rs189009983 | T | C | 0.7055 | 0.00831 | 0.1541 | 4.68E-06 | Cathepsin E | 31.16441532 | 0.000512242 | 20.9598784 | 0.0596 | 0.4341 |
| rs1936839 | C | G | 0.1562 | 0.16937 | 0.0329 | 2.00E-06 | Cathepsin E | 6.653531888 | 0.000550858 | 22.54084866 | 0.0112 | 0.1444 |
| rs57135345 | T | C | 0.2535 | 0.05697 | 0.0542 | 2.95E-06 | Cathepsin E | 10.96113764 | 0.000534606 | 21.87546806 | 0.0177 | 0.1104 |
| rs57689619 | G | A | -0.2078 | 0.08618 | 0.0444 | 2.82E-06 | Cathepsin E | 8.979234524 | 0.000535305 | 21.90408652 | 0.0142 | 0.3204 |
| rs74677283 | T | C | 0.4379 | 0.02151 | 0.0882 | 6.76E-07 | Cathepsin E | 17.83712804 | 0.000602365 | 24.64976142 | 0.0269 | 0.4121 |
| rs8066936 | A | G | -0.1492 | 0.1792 | 0.0325 | 4.27E-06 | Cathepsin E | 6.572637884 | 0.000515058 | 21.07516213 | 0.0106 | 0.1548 |
| rs10745925 | C | T | -0.2874 | 0.29402 | 0.0268 | 9.12E-27 | Cathepsin F | 5.419898316 | 0.002804097 | 115.0016151 | 0.0089 | 0.3114 |
| rs112526544 | G | A | 0.3801 | 0.02831 | 0.0766 | 6.92E-07 | Cathepsin F | 15.4912019 | 0.000601707 | 24.62284323 | 0.0239 | 0.0203002 |
| rs115901379 | G | T | 0.4306 | 0.02042 | 0.0927 | 3.39E-06 | Cathepsin F | 18.74718559 | 0.000527312 | 21.57687684 | 0.0301 | 0.259 |
| rs1260326 | C | T | -0.1642 | 0.40151 | 0.0252 | 6.92E-11 | Cathepsin F | 5.096322298 | 0.001037058 | 42.45660116 | 0.0082 | 0.0258 |
| rs143015877 | T | C | -0.1325 | 0.25734 | 0.0287 | 3.98E-06 | Cathepsin F | 5.804144839 | 0.000520895 | 21.31414731 | 0.0093 | 0.6025 |
| rs1791679 | A | C | 0.2349 | 0.28947 | 0.0269 | 2.51E-18 | Cathepsin F | 5.440121818 | 0.001861063 | 76.25379693 | 0.0088 | 0.5219 |
| rs183683891 | T | C | -0.5242 | 0.01536 | 0.1123 | 3.02E-06 | Cathepsin F | 22.71099183 | 0.00053249 | 21.78886062 | 0.0391 | 0.9942 |
| rs186369051 | T | C | -0.4818 | 0.01504 | 0.1026 | 2.63E-06 | Cathepsin F | 20.74931221 | 0.000538906 | 22.05153723 | 0.0349 | 0.688101 |
| rs58531759 | G | T | -0.3722 | 0.03949 | 0.0747 | 6.31E-07 | Cathepsin F | 15.10695538 | 0.000606676 | 24.82627341 | 0.0279 | 0.2418 |
| rs61866943 | G | T | 0.4969 | 0.01874 | 0.1012 | 9.12E-07 | Cathepsin F | 20.46618319 | 0.000589155 | 24.10887629 | 0.0367 | 0.0363203 |
| rs647400 | A | G | 0.1492 | 0.19253 | 0.0314 | 2.04E-06 | Cathepsin F | 6.350179371 | 0.000551756 | 22.57762992 | 0.0102 | 0.9805 |
| rs7564167 | G | A | -0.2649 | 0.06406 | 0.0526 | 4.90E-07 | Cathepsin F | 10.63756162 | 0.000619772 | 25.36252151 | 0.0163 | 0.1148 |
| rs9796775 | G | A | -0.1172 | 0.49498 | 0.0249 | 2.45E-06 | Cathepsin F | 5.035651794 | 0.000541414 | 22.15422332 | 0.0081 | 0.2381 |
| rs10170044 | G | A | 0.115 | 0.39112 | 0.0251 | 4.79E-06 | Cathepsin G | 5.076098796 | 0.00051302 | 20.99173029 | 0.0083 | 0.678 |
| rs114418234 | C | A | 0.3077 | 0.03719 | 0.065 | 2.24E-06 | Cathepsin G | 13.14527577 | 0.000547645 | 22.40929941 | 0.0219 | 0.7541 |
| rs116142041 | A | G | -0.3356 | 0.03645 | 0.0665 | 4.57E-07 | Cathepsin G | 13.44862829 | 0.000622356 | 25.46833852 | 0.0249 | 0.8306 |
| rs117133380 | A | C | 0.5002 | 0.01556 | 0.1065 | 2.63E-06 | Cathepsin G | 21.53802876 | 0.000539092 | 22.05911878 | 0.0404 | 0.579101 |
| rs147099093 | A | G | 0.4271 | 0.02053 | 0.0885 | 1.38E-06 | Cathepsin G | 17.89779854 | 0.000569159 | 23.29016694 | 0.0325 | 0.1144 |
| rs192289769 | G | C | -0.5468 | 0.01364 | 0.1093 | 5.62E-07 | Cathepsin G | 22.10428679 | 0.000611589 | 25.02745493 | 0.0352 | 0.2599 |
| rs35241999 | G | A | 0.4059 | 0.02374 | 0.0869 | 2.95E-06 | Cathepsin G | 17.57422253 | 0.000533182 | 21.81717673 | 0.028 | 0.3562 |
| rs4702448 | A | C | 0.1275 | 0.33417 | 0.0265 | 1.45E-06 | Cathepsin G | 5.359227813 | 0.000565707 | 23.1488074 | 0.009 | 0.6982 |
| rs497459 | C | T | -0.1752 | 0.13466 | 0.0368 | 1.95E-06 | Cathepsin G | 7.442248434 | 0.000553912 | 22.66587902 | 0.0118 | 0.8735 |
| rs56284011 | C | G | -0.3136 | 0.03943 | 0.0656 | 1.74E-06 | Cathepsin G | 13.26661677 | 0.000558484 | 22.85306365 | 0.0214 | 0.8602 |
| rs62493038 | T | C | -0.3391 | 0.03503 | 0.0725 | 2.88E-06 | Cathepsin G | 14.66203836 | 0.000534633 | 21.87658692 | 0.0242 | 0.6675 |
| rs72773561 | C | T | -0.2529 | 0.05703 | 0.0528 | 1.70E-06 | Cathepsin G | 10.67800862 | 0.000560654 | 22.94192278 | 0.018 | 0.3559 |
| rs77893942 | A | T | -0.376 | 0.03128 | 0.0764 | 8.71E-07 | Cathepsin G | 15.4507549 | 0.000591889 | 24.22082728 | 0.0256 | 0.9595 |
| rs12911554 | T | C | -0.1198 | 0.44973 | 0.0251 | 1.82E-06 | Cathepsin H | 5.076098796 | 0.000556715 | 22.78065428 | 0.0081 | 0.0945802 |
| rs146037740 | A | G | 0.4736 | 0.02292 | 0.0929 | 3.47E-07 | Cathepsin H | 18.7876326 | 0.000635074 | 25.98914304 | 0.0292 | 0.2652 |
| rs147991203 | T | C | 0.3758 | 0.02671 | 0.0773 | 1.17E-06 | Cathepsin H | 15.63276641 | 0.00057758 | 23.63494324 | 0.0287 | 0.6931 |
| rs34593439 | A | G | -1.147 | 0.11066 | 0.0346 | 2.69E-241 | Cathepsin H | 6.997331408 | 0.026167806 | 1098.941662 | 0.0136 | 5.97E-06 |
| rs35628511 | T | C | -0.1253 | 0.3116 | 0.0272 | 4.17E-06 | Cathepsin H | 5.500792321 | 0.000518618 | 21.22092615 | 0.0089 | 0.0420504 |
| rs508807 | C | G | -0.1511 | 0.18975 | 0.032 | 2.40E-06 | Cathepsin H | 6.471520378 | 0.00054488 | 22.29610352 | 0.0103 | 0.6903 |
| rs60018174 | T | C | 0.1956 | 0.1159 | 0.0395 | 7.24E-07 | Cathepsin H | 7.988282966 | 0.000599228 | 24.52130107 | 0.0131 | 0.630901 |
| rs62013235 | A | G | 0.402 | 0.091 | 0.0432 | 1.35E-20 | Cathepsin H | 8.73655251 | 0.002112879 | 86.5933642 | 0.0137 | 0.8583 |
| rs62474230 | C | G | -0.2377 | 0.06195 | 0.0506 | 2.69E-06 | Cathepsin H | 10.2330916 | 0.000539301 | 22.06771313 | 0.017 | 0.654501 |
| rs74342103 | T | A | 0.3766 | 0.02583 | 0.0812 | 3.47E-06 | Cathepsin H | 16.42148296 | 0.000525689 | 21.51040428 | 0.0286 | 0.1564 |
| rs77977134 | C | T | -0.2721 | 0.0493 | 0.0583 | 3.02E-06 | Cathepsin H | 11.79030119 | 0.00053235 | 21.78311449 | 0.0183 | 0.0470197 |
| rs10902420 | G | A | -0.2097 | 0.10091 | 0.0432 | 1.23E-06 | Cathepsin O | 8.73655251 | 0.000575821 | 23.56293403 | 0.0138 | 0.1108 |
| rs146963690 | G | T | 0.6246 | 0.0101 | 0.1322 | 2.29E-06 | Cathepsin O | 26.73546856 | 0.000545522 | 22.3224084 | 0.0422 | 0.8072 |
| rs149159018 | G | A | 0.7034 | 0.01105 | 0.134 | 1.55E-07 | Cathepsin O | 27.09949158 | 0.000673304 | 27.55466474 | 0.0471 | 0.0199402 |
| rs17288007 | G | A | -0.2456 | 0.06381 | 0.0521 | 2.45E-06 | Cathepsin O | 10.53644412 | 0.000543068 | 22.22190458 | 0.0176 | 0.2089 |
| rs181844705 | G | A | 0.4412 | 0.02318 | 0.0905 | 1.10E-06 | Cathepsin O | 18.30226857 | 0.000580805 | 23.7669717 | 0.0302 | 0.3748 |
| rs1870736 | G | C | 0.1222 | 0.42746 | 0.0249 | 9.33E-07 | Cathepsin O | 5.035651794 | 0.000588568 | 24.08483734 | 0.0083 | 0.9926 |
| rs2439803 | G | A | 0.1763 | 0.15304 | 0.0351 | 5.13E-07 | Cathepsin O | 7.098448914 | 0.000616497 | 25.2284397 | 0.0116 | 0.1004 |
| rs4076941 | C | T | -0.1245 | 0.36872 | 0.0262 | 2.14E-06 | Cathepsin O | 5.298557309 | 0.00055183 | 22.58063341 | 0.0085 | 0.605701 |
| rs4297371 | C | A | 0.1453 | 0.20694 | 0.0313 | 3.55E-06 | Cathepsin O | 6.32995587 | 0.00052665 | 21.54976574 | 0.0103 | 0.2638 |
| rs4843804 | A | G | -0.3124 | 0.0336 | 0.0673 | 3.47E-06 | Cathepsin O | 13.61041629 | 0.000526589 | 21.54725354 | 0.022 | 0.1007 |
| rs7140599 | C | T | -0.1219 | 0.42302 | 0.0251 | 1.17E-06 | Cathepsin O | 5.076098796 | 0.000576392 | 23.58630815 | 0.0084 | 0.0159698 |
| rs78943701 | A | G | -0.479 | 0.01536 | 0.1025 | 2.95E-06 | Cathepsin O | 20.72908871 | 0.000533703 | 21.83852469 | 0.0332 | 0.093519 |
| rs9932172 | T | C | 0.1915 | 0.10769 | 0.0401 | 1.74E-06 | Cathepsin O | 8.109623973 | 0.000557334 | 22.80598379 | 0.0133 | 0.9199 |
| rs1022239 | T | A | 0.1157 | 0.41113 | 0.025 | 3.72E-06 | Cathepsin S | 5.055875295 | 0.000523441 | 21.418384 | 0.0084 | 0.4094 |
| rs10516855 | C | T | -0.1969 | 0.09552 | 0.0421 | 2.95E-06 | Cathepsin S | 8.514093997 | 0.000534569 | 21.87395129 | 0.0139 | 0.1947 |
| rs1060435 | G | A | -0.1291 | 0.39793 | 0.0251 | 2.75E-07 | Cathepsin S | 5.076098796 | 0.000646447 | 26.45483405 | 0.0084 | 0.8606 |
| rs113108135 | C | G | 0.2349 | 0.06933 | 0.0491 | 1.70E-06 | Cathepsin S | 9.92973908 | 0.000559331 | 22.88774727 | 0.0175 | 0.4807 |
| rs116623438 | C | T | -0.43 | 0.02023 | 0.0876 | 9.12E-07 | Cathepsin S | 17.71578703 | 0.000588819 | 24.0950981 | 0.0307 | 0.660601 |
| rs118010753 | C | T | 0.5018 | 0.01437 | 0.1081 | 3.47E-06 | Cathepsin S | 21.86160478 | 0.000526611 | 21.54814682 | 0.041 | 0.8008 |
| rs12804405 | A | G | 0.612 | 0.01019 | 0.129 | 2.09E-06 | Cathepsin S | 26.08831652 | 0.000550038 | 22.50730124 | 0.0408 | 0.1157 |
| rs13150189 | A | G | 0.1556 | 0.21113 | 0.0306 | 3.47E-07 | Cathepsin S | 6.188391361 | 0.000631845 | 25.85689265 | 0.0102 | 0.3336 |
| rs13196989 | T | C | -0.1758 | 0.13099 | 0.0374 | 2.57E-06 | Cathepsin S | 7.563589442 | 0.000539969 | 22.09502702 | 0.0123 | 0.684301 |
| rs13212873 | C | T | 0.5422 | 0.0116 | 0.117 | 3.55E-06 | Cathepsin S | 23.66149638 | 0.000524841 | 21.47569874 | 0.0363 | 0.4913 |
| rs13411643 | C | T | 0.1831 | 0.13712 | 0.0364 | 5.01E-07 | Cathepsin S | 7.36135443 | 0.000618321 | 25.30311104 | 0.0116 | 0.5683 |
| rs2470994 | C | T | 0.1304 | 0.28088 | 0.0281 | 3.39E-06 | Cathepsin S | 5.682803832 | 0.000526287 | 21.53488431 | 0.0091 | 0.0467897 |
| rs41271951 | G | A | -0.8605 | 0.08349 | 0.0419 | 7.08E-94 | Cathepsin S | 8.473646995 | 0.010207663 | 421.7680749 | 0.0154 | 0.621199 |
| rs4313886 | C | T | 0.1687 | 0.13446 | 0.0365 | 3.89E-06 | Cathepsin S | 7.381577931 | 0.000522067 | 21.36212423 | 0.0122 | 0.8901 |
| rs4581957 | A | G | -0.1775 | 0.13414 | 0.0365 | 1.15E-06 | Cathepsin S | 7.381577931 | 0.000577921 | 23.64890223 | 0.0123 | 0.2181 |
| rs529565 | C | T | -0.1315 | 0.31269 | 0.0266 | 7.76E-07 | Cathepsin S | 5.379451314 | 0.000597224 | 24.43927017 | 0.0086 | 0.3162 |
| rs6657328 | C | G | -0.1291 | 0.2816 | 0.0283 | 4.90E-06 | Cathepsin S | 5.723250834 | 0.000508589 | 20.81036097 | 0.0092 | 0.122 |
| rs73099998 | T | C | -0.146 | 0.20811 | 0.0308 | 2.19E-06 | Cathepsin S | 6.228838364 | 0.000549129 | 22.47006241 | 0.0101 | 0.01837 |
| rs74804137 | C | T | -0.5178 | 0.01757 | 0.0987 | 1.58E-07 | Cathepsin S | 19.96059567 | 0.000672521 | 27.52262082 | 0.0359 | 0.7694 |
| rs7614425 | A | G | -0.2082 | 0.09254 | 0.0436 | 1.82E-06 | Cathepsin S | 8.817446515 | 0.000557256 | 22.80281542 | 0.0142 | 0.8911 |
| rs77792819 | G | A | 0.3147 | 0.04096 | 0.0661 | 1.95E-06 | Cathepsin S | 13.36773428 | 0.000553935 | 22.66681849 | 0.0204 | 0.0601105 |
| rs78767885 | C | T | 0.2238 | 0.08725 | 0.0477 | 2.69E-06 | Cathepsin S | 9.646610063 | 0.00053797 | 22.0132115 | 0.0167 | 0.8526 |
| rs989576 | T | C | 0.1307 | 0.29843 | 0.0278 | 2.57E-06 | Cathepsin S | 5.622133328 | 0.000540176 | 22.10352725 | 0.0091 | 0.4292 |
| rs10817163 | A | T | 0.1983 | 0.22045 | 0.0304 | 7.08E-11 | Cathepsin L2 | 6.147944359 | 0.001039334 | 42.54987232 | 0.0099 | 0.3042 |
| rs114113108 | C | G | -0.5922 | 0.0129 | 0.1172 | 4.37E-07 | Cathepsin L2 | 23.70194338 | 0.000623906 | 25.53180876 | 0.0351 | 0.0810401 |
| rs116407656 | C | T | 0.4014 | 0.02439 | 0.0856 | 2.75E-06 | Cathepsin L2 | 17.31131701 | 0.000537381 | 21.98908747 | 0.0312 | 0.2768 |
| rs117714361 | G | A | -0.3452 | 0.02685 | 0.0756 | 4.90E-06 | Cathepsin L2 | 15.28896689 | 0.000509548 | 20.84961227 | 0.0278 | 0.4263 |
| rs13068566 | G | A | 0.1157 | 0.46617 | 0.0247 | 2.88E-06 | Cathepsin L2 | 4.995204792 | 0.000536227 | 21.94182825 | 0.0082 | 0.2432 |
| rs148608463 | A | G | -0.1178 | 0.35415 | 0.0257 | 4.57E-06 | Cathepsin L2 | 5.197439803 | 0.000513464 | 21.00991688 | 0.0085 | 0.0411604 |
| rs151179824 | A | G | 0.4449 | 0.02158 | 0.0943 | 2.34E-06 | Cathepsin L2 | 19.07076161 | 0.000543968 | 22.25878354 | 0.0361 | 0.1821 |
| rs1523319 | G | C | -0.1292 | 0.28541 | 0.0279 | 3.55E-06 | Cathepsin L2 | 5.642356829 | 0.00052408 | 21.44453437 | 0.0092 | 0.1277 |
| rs2302837 | G | A | -0.2541 | 0.07506 | 0.0498 | 3.31E-07 | Cathepsin L2 | 10.07130359 | 0.000636184 | 26.03458412 | 0.016 | 0.1762 |
| rs7669728 | C | T | -0.1301 | 0.43633 | 0.0254 | 3.09E-07 | Cathepsin L2 | 5.1367693 | 0.000641087 | 26.23536797 | 0.0082 | 0.3242 |
| rs7898416 | G | A | -0.2277 | 0.0705 | 0.0489 | 3.24E-06 | Cathepsin L2 | 9.889292077 | 0.000529891 | 21.68244947 | 0.0159 | 0.3466 |
| rs10745925 | C | T | -0.3624 | 0.29402 | 0.0266 | 2.29E-42 | Cathepsin Z | 5.379451314 | 0.004518092 | 185.615015 | 0.0089 | 0.3114 |
| rs10761760 | C | A | 0.1321 | 0.44944 | 0.0258 | 3.09E-07 | Cathepsin Z | 5.217663305 | 0.000640614 | 26.21598762 | 0.0081 | 0.00144202 |
| rs1135945 | A | G | 0.1385 | 0.20839 | 0.0301 | 4.17E-06 | Cathepsin Z | 6.087273855 | 0.000517428 | 21.17222768 | 0.0101 | 0.3998 |
| rs114675081 | G | A | 0.5153 | 0.01467 | 0.1075 | 1.66E-06 | Cathepsin Z | 21.74026377 | 0.000561524 | 22.97753077 | 0.0358 | 0.8025 |
| rs116920068 | A | G | -0.3498 | 0.0289 | 0.0749 | 3.02E-06 | Cathepsin Z | 15.14740238 | 0.000533032 | 21.81101994 | 0.0259 | 0.1809 |
| rs148201372 | T | A | -0.5187 | 0.01546 | 0.1063 | 1.05E-06 | Cathepsin Z | 21.49758175 | 0.000581864 | 23.81036028 | 0.0406 | 0.097699 |
| rs148370779 | T | C | -1.7632 | 0.01316 | 0.1105 | 2.57E-57 | Cathepsin Z | 22.3469688 | 0.006187166 | 254.6118417 | 0.0459 | 0.2522 |
| rs298724 | C | T | 0.2482 | 0.05507 | 0.0538 | 3.98E-06 | Cathepsin Z | 10.88024364 | 0.000520142 | 21.28330178 | 0.0185 | 0.3071 |
| rs36128387 | T | C | 0.439 | 0.02261 | 0.0914 | 1.55E-06 | Cathepsin Z | 18.48428008 | 0.000563768 | 23.06941858 | 0.0316 | 0.785301 |
| rs4761709 | G | A | 0.1218 | 0.40882 | 0.0249 | 1.00E-06 | Cathepsin Z | 5.035651794 | 0.000584723 | 23.92742053 | 0.0083 | 0.9519 |
| rs67845377 | T | C | 0.1675 | 0.13855 | 0.0362 | 3.72E-06 | Cathepsin Z | 7.320907427 | 0.000523231 | 21.40979366 | 0.0128 | 0.0400904 |
| rs7656806 | C | A | -0.1276 | 0.48408 | 0.0245 | 1.86E-07 | Cathepsin Z | 4.954757789 | 0.000662811 | 27.1249646 | 0.0081 | 0.8438 |
| rs770140 | G | A | -0.1241 | 0.35805 | 0.0265 | 2.82E-06 | Cathepsin Z | 5.359227813 | 0.000535954 | 21.93066572 | 0.0086 | 0.3529 |
| rs10016148 | A | T | -0.0896 | 0.9016 | 0.0193 | 3.39E-06 | Cathepsin L1 | 2.672614495 | 0.001122796 | 21.55268598 | 0.0134 | 0.4058 |
| rs1031153 | T | C | 0.1061 | 0.2635 | 0.0128 | 1.25E-16 | Cathepsin L1 | 1.607967363 | 0.004335552 | 68.70855713 | 0.0093 | 0.8524 |
| rs10469365 | A | G | 0.096 | 0.8978 | 0.0196 | 9.10E-07 | Cathepsin L1 | 2.387904286 | 0.001613859 | 23.99000416 | 0.0146 | 0.5919 |
| rs113096165 | T | C | -0.0684 | 0.208 | 0.014 | 1.10E-06 | Cathepsin L1 | 1.841838212 | 0.001377402 | 23.87020408 | 0.01 | 0.1013 |
| rs11586939 | T | G | -0.2745 | 0.0235 | 0.0505 | 5.60E-08 | Cathepsin L1 | 5.903534238 | 0.002157675 | 29.54622096 | 0.0309 | 0.9073 |
| rs12620053 | A | C | 0.0578 | 0.5575 | 0.0122 | 2.31E-06 | Cathepsin L1 | 1.486298516 | 0.001510239 | 22.44584789 | 0.0083 | 0.751 |
| rs143645865 | T | C | -0.1494 | 0.0346 | 0.0324 | 4.07E-06 | Cathepsin L1 | 4.375439763 | 0.00116466 | 21.26234568 | 0.0245 | 0.9607 |
| rs148670635 | C | G | 0.2503 | 0.9812 | 0.0538 | 3.29E-06 | Cathepsin L1 | 6.128242733 | 0.001665684 | 21.64497796 | 0.0405 | 0.939 |
| rs150011041 | T | G | -0.1849 | 0.9729 | 0.04 | 3.71E-06 | Cathepsin L1 | 5.407846152 | 0.001167791 | 21.36750625 | 0.0393 | 0.6893 |
| rs150370599 | T | C | 0.1566 | 0.0745 | 0.0211 | 1.16E-13 | Cathepsin L1 | 2.921873878 | 0.002864574 | 55.08312931 | 0.0153 | 0.4236 |
| rs1580289 | A | G | 0.048 | 0.4671 | 0.0103 | 3.50E-06 | Cathepsin L1 | 1.42628039 | 0.001131426 | 21.71740975 | 0.0082 | 0.9579 |
| rs17151689 | A | C | -0.1197 | 0.0442 | 0.0256 | 2.87E-06 | Cathepsin L1 | 3.545022336 | 0.00113894 | 21.8629303 | 0.0198 | 0.742 |
| rs181283433 | C | G | 0.3521 | 0.9909 | 0.0768 | 4.50E-06 | Cathepsin L1 | 8.67806017 | 0.001643767 | 21.0188819 | 0.0545 | 0.9465 |
| rs184593554 | C | G | 0.2672 | 0.0122 | 0.0585 | 4.88E-06 | Cathepsin L1 | 7.497819733 | 0.001268541 | 20.86225144 | 0.0437 | 0.5758 |
| rs2274611 | T | C | -0.1179 | 0.4415 | 0.0104 | 4.85E-30 | Cathepsin L1 | 1.440127772 | 0.006658394 | 128.5171043 | 0.0082 | 0.7147 |
| rs2921189 | A | G | 0.0534 | 0.3692 | 0.0106 | 4.08E-07 | Cathepsin L1 | 1.467822537 | 0.001321923 | 25.37878248 | 0.0084 | 0.0244698 |
| rs3001922 | A | G | 0.0821 | 0.1022 | 0.0178 | 4.01E-06 | Cathepsin L1 | 2.464898343 | 0.001108286 | 21.27386062 | 0.0143 | 0.6524 |
| rs3129757 | A | C | 0.107 | 0.1514 | 0.0146 | 2.66E-13 | Cathepsin L1 | 2.021401504 | 0.002794425 | 53.71082755 | 0.0121 | 0.0579696 |
| rs35049778 | A | G | 0.0711 | 0.2771 | 0.0118 | 1.67E-09 | Cathepsin L1 | 1.633991126 | 0.001890007 | 36.30573111 | 0.0091 | 0.2288 |
| rs554689923 | C | G | 0.3594 | 0.9862 | 0.0755 | 1.93E-06 | Cathepsin L1 | 6.920924848 | 0.002690057 | 22.66012192 | 0.0423 | 0.0652695 |
| rs6575449 | T | C | -0.2042 | 0.1826 | 0.0145 | 7.09E-45 | Cathepsin L1 | 1.821525528 | 0.012412848 | 198.3240904 | 0.0107 | 0.1518 |
| rs73033060 | T | G | 0.1463 | 0.0453 | 0.0307 | 1.84E-06 | Cathepsin L1 | 3.740111841 | 0.001527967 | 22.70972636 | 0.0202 | 0.3064 |
| rs76438938 | T | C | 0.1518 | 0.0306 | 0.0307 | 7.53E-07 | Cathepsin L1 | 4.251478945 | 0.001273372 | 24.44932042 | 0.0248 | 0.3663 |
| rs76453951 | T | C | 0.0844 | 0.117 | 0.0161 | 1.65E-07 | Cathepsin L1 | 2.229544835 | 0.001431119 | 27.48103854 | 0.0133 | 0.2471 |
| rs76904798 | T | C | 0.1095 | 0.13 | 0.0154 | 1.01E-12 | Cathepsin L1 | 2.132663705 | 0.002629573 | 50.55764041 | 0.0117 | 0.5894 |
| rs8176396 | T | C | -0.1971 | 0.0316 | 0.035 | 1.85E-08 | Cathepsin L1 | 4.396925062 | 0.002005664 | 31.71298776 | 0.0257 | 0.6004 |
| rs887945 | A | G | -0.1668 | 0.7645 | 0.0121 | 3.80E-43 | Cathepsin L1 | 1.675533273 | 0.009814045 | 190.0296428 | 0.0104 | 0.1003 |
| rs9477379 | T | C | 0.051 | 0.587 | 0.0108 | 2.29E-06 | Cathepsin L1 | 1.495517302 | 0.001161711 | 22.29938272 | 0.0082 | 0.1334 |
| rs9497486 | T | G | 0.1398 | 0.0731 | 0.0212 | 4.54E-11 | Cathepsin L1 | 2.935721622 | 0.002262799 | 43.48531506 | 0.0168 | 0.03992 |
| rs9497576 | T | G | 0.1346 | 0.0749 | 0.0223 | 1.69E-09 | Cathepsin L1 | 2.716850285 | 0.002448795 | 36.43178025 | 0.0154 | 0.98 |
| rs9901673 | A | C | -0.0701 | 0.1634 | 0.0139 | 4.46E-07 | Cathepsin L1 | 1.924836346 | 0.001324701 | 25.43351793 | 0.0109 | 0.685799 |

| Table S2. SNPs information of ten cathepsins with amyotrophic lateral sclerosis | | | | | | | | | | | | |
| --- | --- | --- | --- | --- | --- | --- | --- | --- | --- | --- | --- | --- |
| **Ten cathepsins** | | | | | | | | | |  | **Amyotrophic lateral sclerosis** | |
| SNP | effect_allele | other_allele | beta | eaf | se | pval | exposure | sd | R2 | F | se | pval |
| rs113646963 | T | C | 0.1996 | 0.09708 | 0.0427 | 2.95E-06 | Cathepsin B | 8.635435004 | 0.000534001 | 21.85069846 | 0.0209 | 0.9182 |
| rs11563944 | G | A | 0.1291 | 0.26799 | 0.0278 | 3.55E-06 | Cathepsin B | 5.622133328 | 0.000527039 | 21.56566689 | 0.0127 | 0.3157 |
| rs117486267 | C | T | -0.4003 | 0.02486 | 0.0834 | 1.58E-06 | Cathepsin B | 16.86639998 | 0.000562993 | 23.0376979 | 0.0424 | 0.1101 |
| rs143557119 | A | G | 0.4327 | 0.02199 | 0.0946 | 4.79E-06 | Cathepsin B | 19.13143212 | 0.000511303 | 20.92143732 | 0.0462 | 0.00558599 |
| rs147881440 | A | G | -0.2331 | 0.06428 | 0.051 | 4.79E-06 | Cathepsin B | 10.3139856 | 0.000510541 | 20.89027682 | 0.0294 | 0.00770992 |
| rs148930853 | C | T | 0.5516 | 0.01211 | 0.1181 | 3.02E-06 | Cathepsin B | 23.88395489 | 0.000533121 | 21.81467363 | 0.0632 | 0.5782 |
| rs150370599 | T | C | 0.2183 | 0.08341 | 0.0457 | 1.78E-06 | Cathepsin B | 9.24214004 | 0.000557624 | 22.81786841 | 0.0254 | 0.1218 |
| rs1692819 | A | G | 0.4246 | 0.29182 | 0.0274 | 5.25E-54 | Cathepsin B | 5.541239323 | 0.005837472 | 240.1368746 | 0.0133 | 0.1539 |
| rs36021960 | A | G | 0.1747 | 0.12637 | 0.0374 | 3.02E-06 | Cathepsin B | 7.563589442 | 0.000533236 | 21.81939003 | 0.016 | 0.6332 |
| rs7249773 | A | G | 0.1749 | 0.15121 | 0.0351 | 6.03E-07 | Cathepsin B | 7.098448914 | 0.000606751 | 24.82935203 | 0.0154 | 0.9707 |
| rs72863882 | A | G | -0.2569 | 0.08353 | 0.0475 | 6.46E-08 | Cathepsin B | 9.606163061 | 0.000714725 | 29.25101828 | 0.0262 | 0.1512 |
| rs75773690 | A | G | -0.3945 | 0.02192 | 0.0857 | 4.17E-06 | Cathepsin B | 17.33154051 | 0.000517864 | 21.19006902 | 0.038 | 0.2889 |
| rs76089522 | A | G | -0.1971 | 0.10475 | 0.0421 | 2.88E-06 | Cathepsin B | 8.514093997 | 0.000535655 | 21.91841053 | 0.0198 | 0.8697 |
| rs9905973 | A | G | -0.1405 | 0.271 | 0.0276 | 3.55E-07 | Cathepsin B | 5.581686326 | 0.000633239 | 25.91400179 | 0.013 | 0.00841008 |
| rs10401027 | A | G | -0.1225 | 0.45246 | 0.0259 | 2.24E-06 | Cathepsin E | 5.237886806 | 0.000546693 | 22.37034332 | 0.0181 | 0.0987393 |
| rs10900907 | A | G | 0.1188 | 0.41707 | 0.0255 | 3.24E-06 | Cathepsin E | 5.156992801 | 0.000530433 | 21.70463668 | 0.0116 | 0.4933 |
| rs112918835 | T | C | 0.7062 | 0.00706 | 0.1526 | 3.72E-06 | Cathepsin E | 30.8610628 | 0.000523393 | 21.41639455 | 0.0625 | 0.747699 |
| rs13089837 | C | T | -0.1152 | 0.49848 | 0.025 | 4.17E-06 | Cathepsin E | 5.055875295 | 0.000518929 | 21.233664 | 0.0113 | 0.9085 |
| rs1936839 | C | G | 0.1562 | 0.16937 | 0.0329 | 2.00E-06 | Cathepsin E | 6.653531888 | 0.000550858 | 22.54084866 | 0.0154 | 0.1884 |
| rs57135345 | T | C | 0.2535 | 0.05697 | 0.0542 | 2.95E-06 | Cathepsin E | 10.96113764 | 0.000534606 | 21.87546806 | 0.0241 | 0.0223702 |
| rs57689619 | G | A | -0.2078 | 0.08618 | 0.0444 | 2.82E-06 | Cathepsin E | 8.979234524 | 0.000535305 | 21.90408652 | 0.0193 | 0.676299 |
| rs74677283 | T | C | 0.4379 | 0.02151 | 0.0882 | 6.76E-07 | Cathepsin E | 17.83712804 | 0.000602365 | 24.64976142 | 0.0385 | 0.0771792 |
| rs8066936 | A | G | -0.1492 | 0.1792 | 0.0325 | 4.27E-06 | Cathepsin E | 6.572637884 | 0.000515058 | 21.07516213 | 0.0155 | 0.783201 |
| rs10745925 | C | T | -0.2874 | 0.29402 | 0.0268 | 9.12E-27 | Cathepsin F | 5.419898316 | 0.002804097 | 115.0016151 | 0.0121 | 0.971 |
| rs112526544 | G | A | 0.3801 | 0.02831 | 0.0766 | 6.92E-07 | Cathepsin F | 15.4912019 | 0.000601707 | 24.62284323 | 0.0352 | 0.3794 |
| rs115901379 | G | T | 0.4306 | 0.02042 | 0.0927 | 3.39E-06 | Cathepsin F | 18.74718559 | 0.000527312 | 21.57687684 | 0.0446 | 0.8895 |
| rs1260326 | C | T | -0.1642 | 0.40151 | 0.0252 | 6.92E-11 | Cathepsin F | 5.096322298 | 0.001037058 | 42.45660116 | 0.0111 | 0.1795 |
| rs143015877 | T | C | -0.1325 | 0.25734 | 0.0287 | 3.98E-06 | Cathepsin F | 5.804144839 | 0.000520895 | 21.31414731 | 0.0134 | 0.8333 |
| rs1791679 | A | C | 0.2349 | 0.28947 | 0.0269 | 2.51E-18 | Cathepsin F | 5.440121818 | 0.001861063 | 76.25379693 | 0.0119 | 0.318 |
| rs647400 | A | G | 0.1492 | 0.19253 | 0.0314 | 2.04E-06 | Cathepsin F | 6.350179371 | 0.000551756 | 22.57762992 | 0.0139 | 0.9533 |
| rs7564167 | G | A | -0.2649 | 0.06406 | 0.0526 | 4.90E-07 | Cathepsin F | 10.63756162 | 0.000619772 | 25.36252151 | 0.0228 | 0.0830998 |
| rs9796775 | G | A | -0.1172 | 0.49498 | 0.0249 | 2.45E-06 | Cathepsin F | 5.035651794 | 0.000541414 | 22.15422332 | 0.0113 | 0.673701 |
| rs10170044 | G | A | 0.115 | 0.39112 | 0.0251 | 4.79E-06 | Cathepsin G | 5.076098796 | 0.00051302 | 20.99173029 | 0.0115 | 0.3939 |
| rs114418234 | C | A | 0.3077 | 0.03719 | 0.065 | 2.24E-06 | Cathepsin G | 13.14527577 | 0.000547645 | 22.40929941 | 0.0466 | 0.7011 |
| rs116142041 | A | G | -0.3356 | 0.03645 | 0.0665 | 4.57E-07 | Cathepsin G | 13.44862829 | 0.000622356 | 25.46833852 | 0.0389 | 0.9036 |
| rs147099093 | A | G | 0.4271 | 0.02053 | 0.0885 | 1.38E-06 | Cathepsin G | 17.89779854 | 0.000569159 | 23.29016694 | 0.0459 | 0.3335 |
| rs192289769 | G | C | -0.5468 | 0.01364 | 0.1093 | 5.62E-07 | Cathepsin G | 22.10428679 | 0.000611589 | 25.02745493 | 0.0505 | 0.1242 |
| rs497459 | C | T | -0.1752 | 0.13466 | 0.0368 | 1.95E-06 | Cathepsin G | 7.442248434 | 0.000553912 | 22.66587902 | 0.0159 | 0.9342 |
| rs56284011 | C | G | -0.3136 | 0.03943 | 0.0656 | 1.74E-06 | Cathepsin G | 13.26661677 | 0.000558484 | 22.85306365 | 0.0311 | 0.2467 |
| rs62493038 | T | C | -0.3391 | 0.03503 | 0.0725 | 2.88E-06 | Cathepsin G | 14.66203836 | 0.000534633 | 21.87658692 | 0.0359 | 0.8193 |
| rs72773561 | C | T | -0.2529 | 0.05703 | 0.0528 | 1.70E-06 | Cathepsin G | 10.67800862 | 0.000560654 | 22.94192278 | 0.0257 | 0.4102 |
| rs77893942 | A | T | -0.376 | 0.03128 | 0.0764 | 8.71E-07 | Cathepsin G | 15.4507549 | 0.000591889 | 24.22082728 | 0.0384 | 0.5802 |
| rs12911554 | T | C | -0.1198 | 0.44973 | 0.0251 | 1.82E-06 | Cathepsin H | 5.076098796 | 0.000556715 | 22.78065428 | 0.011 | 0.7511 |
| rs35628511 | T | C | -0.1253 | 0.3116 | 0.0272 | 4.17E-06 | Cathepsin H | 5.500792321 | 0.000518618 | 21.22092615 | 0.0122 | 0.288 |
| rs508807 | C | G | -0.1511 | 0.18975 | 0.032 | 2.40E-06 | Cathepsin H | 6.471520378 | 0.00054488 | 22.29610352 | 0.0199 | 0.966 |
| rs60018174 | T | C | 0.1956 | 0.1159 | 0.0395 | 7.24E-07 | Cathepsin H | 7.988282966 | 0.000599228 | 24.52130107 | 0.027 | 0.381 |
| rs62013235 | A | G | 0.402 | 0.091 | 0.0432 | 1.35E-20 | Cathepsin H | 8.73655251 | 0.002112879 | 86.5933642 | 0.019 | 0.2074 |
| rs62474230 | C | G | -0.2377 | 0.06195 | 0.0506 | 2.69E-06 | Cathepsin H | 10.2330916 | 0.000539301 | 22.06771313 | 0.0233 | 0.9246 |
| rs74342103 | T | A | 0.3766 | 0.02583 | 0.0812 | 3.47E-06 | Cathepsin H | 16.42148296 | 0.000525689 | 21.51040428 | 0.0361 | 0.2128 |
| rs77977134 | C | T | -0.2721 | 0.0493 | 0.0583 | 3.02E-06 | Cathepsin H | 11.79030119 | 0.00053235 | 21.78311449 | 0.0262 | 0.0378303 |
| rs10902420 | G | A | -0.2097 | 0.10091 | 0.0432 | 1.23E-06 | Cathepsin O | 8.73655251 | 0.000575821 | 23.56293403 | 0.0215 | 0.7845 |
| rs146963690 | G | T | 0.6246 | 0.0101 | 0.1322 | 2.29E-06 | Cathepsin O | 26.73546856 | 0.000545522 | 22.3224084 | 0.0563 | 0.2342 |
| rs17288007 | G | A | -0.2456 | 0.06381 | 0.0521 | 2.45E-06 | Cathepsin O | 10.53644412 | 0.000543068 | 22.22190458 | 0.0356 | 0.7752 |
| rs1870736 | G | C | 0.1222 | 0.42746 | 0.0249 | 9.33E-07 | Cathepsin O | 5.035651794 | 0.000588568 | 24.08483734 | 0.0113 | 0.8064 |
| rs2439803 | G | A | 0.1763 | 0.15304 | 0.0351 | 5.13E-07 | Cathepsin O | 7.098448914 | 0.000616497 | 25.2284397 | 0.0156 | 0.9306 |
| rs4076941 | C | T | -0.1245 | 0.36872 | 0.0262 | 2.14E-06 | Cathepsin O | 5.298557309 | 0.00055183 | 22.58063341 | 0.0115 | 0.8928 |
| rs4297371 | C | A | 0.1453 | 0.20694 | 0.0313 | 3.55E-06 | Cathepsin O | 6.32995587 | 0.00052665 | 21.54976574 | 0.0138 | 0.2565 |
| rs78943701 | A | G | -0.479 | 0.01536 | 0.1025 | 2.95E-06 | Cathepsin O | 20.72908871 | 0.000533703 | 21.83852469 | 0.0565 | 0.1025 |
| rs9932172 | T | C | 0.1915 | 0.10769 | 0.0401 | 1.74E-06 | Cathepsin O | 8.109623973 | 0.000557334 | 22.80598379 | 0.0181 | 0.4456 |
| rs1022239 | T | A | 0.1157 | 0.41113 | 0.025 | 3.72E-06 | Cathepsin S | 5.055875295 | 0.000523441 | 21.418384 | 0.0115 | 0.650801 |
| rs10516855 | C | T | -0.1969 | 0.09552 | 0.0421 | 2.95E-06 | Cathepsin S | 8.514093997 | 0.000534569 | 21.87395129 | 0.0286 | 0.5798 |
| rs1060435 | G | A | -0.1291 | 0.39793 | 0.0251 | 2.75E-07 | Cathepsin S | 5.076098796 | 0.000646447 | 26.45483405 | 0.0114 | 0.673899 |
| rs113108135 | C | G | 0.2349 | 0.06933 | 0.0491 | 1.70E-06 | Cathepsin S | 9.92973908 | 0.000559331 | 22.88774727 | 0.0251 | 0.7957 |
| rs118010753 | C | T | 0.5018 | 0.01437 | 0.1081 | 3.47E-06 | Cathepsin S | 21.86160478 | 0.000526611 | 21.54814682 | 0.0587 | 0.7452 |
| rs12804405 | A | G | 0.612 | 0.01019 | 0.129 | 2.09E-06 | Cathepsin S | 26.08831652 | 0.000550038 | 22.50730124 | 0.0545 | 0.911 |
| rs13150189 | A | G | 0.1556 | 0.21113 | 0.0306 | 3.47E-07 | Cathepsin S | 6.188391361 | 0.000631845 | 25.85689265 | 0.014 | 0.8999 |
| rs13196989 | T | C | -0.1758 | 0.13099 | 0.0374 | 2.57E-06 | Cathepsin S | 7.563589442 | 0.000539969 | 22.09502702 | 0.017 | 0.3247 |
| rs13212873 | C | T | 0.5422 | 0.0116 | 0.117 | 3.55E-06 | Cathepsin S | 23.66149638 | 0.000524841 | 21.47569874 | 0.0516 | 0.8282 |
| rs13411643 | C | T | 0.1831 | 0.13712 | 0.0364 | 5.01E-07 | Cathepsin S | 7.36135443 | 0.000618321 | 25.30311104 | 0.0166 | 0.6595 |
| rs2470994 | C | T | 0.1304 | 0.28088 | 0.0281 | 3.39E-06 | Cathepsin S | 5.682803832 | 0.000526287 | 21.53488431 | 0.0126 | 0.6008 |
| rs41271951 | G | A | -0.8605 | 0.08349 | 0.0419 | 7.08E-94 | Cathepsin S | 8.473646995 | 0.010207663 | 421.7680749 | 0.0219 | 0.0642703 |
| rs4313886 | C | T | 0.1687 | 0.13446 | 0.0365 | 3.89E-06 | Cathepsin S | 7.381577931 | 0.000522067 | 21.36212423 | 0.0178 | 0.2666 |
| rs4581957 | A | G | -0.1775 | 0.13414 | 0.0365 | 1.15E-06 | Cathepsin S | 7.381577931 | 0.000577921 | 23.64890223 | 0.0171 | 0.4327 |
| rs529565 | C | T | -0.1315 | 0.31269 | 0.0266 | 7.76E-07 | Cathepsin S | 5.379451314 | 0.000597224 | 24.43927017 | 0.0116 | 0.857 |
| rs6657328 | C | G | -0.1291 | 0.2816 | 0.0283 | 4.90E-06 | Cathepsin S | 5.723250834 | 0.000508589 | 20.81036097 | 0.0125 | 0.4384 |
| rs73099998 | T | C | -0.146 | 0.20811 | 0.0308 | 2.19E-06 | Cathepsin S | 6.228838364 | 0.000549129 | 22.47006241 | 0.0139 | 0.8763 |
| rs74804137 | C | T | -0.5178 | 0.01757 | 0.0987 | 1.58E-07 | Cathepsin S | 19.96059567 | 0.000672521 | 27.52262082 | 0.0517 | 0.6916 |
| rs7614425 | A | G | -0.2082 | 0.09254 | 0.0436 | 1.82E-06 | Cathepsin S | 8.817446515 | 0.000557256 | 22.80281542 | 0.0195 | 0.740399 |
| rs77792819 | G | A | 0.3147 | 0.04096 | 0.0661 | 1.95E-06 | Cathepsin S | 13.36773428 | 0.000553935 | 22.66681849 | 0.0293 | 0.3289 |
| rs78767885 | C | T | 0.2238 | 0.08725 | 0.0477 | 2.69E-06 | Cathepsin S | 9.646610063 | 0.00053797 | 22.0132115 | 0.0266 | 0.7536 |
| rs989576 | T | C | 0.1307 | 0.29843 | 0.0278 | 2.57E-06 | Cathepsin S | 5.622133328 | 0.000540176 | 22.10352725 | 0.0131 | 0.2776 |
| rs114113108 | C | G | -0.5922 | 0.0129 | 0.1172 | 4.37E-07 | Cathepsin L2 | 23.70194338 | 0.000623906 | 25.53180876 | 0.0477 | 0.2694 |
| rs116407656 | C | T | 0.4014 | 0.02439 | 0.0856 | 2.75E-06 | Cathepsin L2 | 17.31131701 | 0.000537381 | 21.98908747 | 0.0448 | 0.9958 |
| rs117714361 | G | A | -0.3452 | 0.02685 | 0.0756 | 4.90E-06 | Cathepsin L2 | 15.28896689 | 0.000509548 | 20.84961227 | 0.0418 | 0.718199 |
| rs13068566 | G | A | 0.1157 | 0.46617 | 0.0247 | 2.88E-06 | Cathepsin L2 | 4.995204792 | 0.000536227 | 21.94182825 | 0.011 | 0.8363 |
| rs148608463 | A | G | -0.1178 | 0.35415 | 0.0257 | 4.57E-06 | Cathepsin L2 | 5.197439803 | 0.000513464 | 21.00991688 | 0.0115 | 0.4584 |
| rs1523319 | G | C | -0.1292 | 0.28541 | 0.0279 | 3.55E-06 | Cathepsin L2 | 5.642356829 | 0.00052408 | 21.44453437 | 0.0127 | 0.2275 |
| rs2302837 | G | A | -0.2541 | 0.07506 | 0.0498 | 3.31E-07 | Cathepsin L2 | 10.07130359 | 0.000636184 | 26.03458412 | 0.0277 | 0.898 |
| rs7669728 | C | T | -0.1301 | 0.43633 | 0.0254 | 3.09E-07 | Cathepsin L2 | 5.1367693 | 0.000641087 | 26.23536797 | 0.0113 | 0.821 |
| rs7898416 | G | A | -0.2277 | 0.0705 | 0.0489 | 3.24E-06 | Cathepsin L2 | 9.889292077 | 0.000529891 | 21.68244947 | 0.0221 | 0.8185 |
| rs10745925 | C | T | -0.3624 | 0.29402 | 0.0266 | 2.29E-42 | Cathepsin Z | 5.379451314 | 0.004518092 | 185.615015 | 0.0121 | 0.971 |
| rs10761760 | C | A | 0.1321 | 0.44944 | 0.0258 | 3.09E-07 | Cathepsin Z | 5.217663305 | 0.000640614 | 26.21598762 | 0.011 | 0.5303 |
| rs1135945 | A | G | 0.1385 | 0.20839 | 0.0301 | 4.17E-06 | Cathepsin Z | 6.087273855 | 0.000517428 | 21.17222768 | 0.0159 | 0.6307 |
| rs114675081 | G | A | 0.5153 | 0.01467 | 0.1075 | 1.66E-06 | Cathepsin Z | 21.74026377 | 0.000561524 | 22.97753077 | 0.0543 | 0.3111 |
| rs116920068 | A | G | -0.3498 | 0.0289 | 0.0749 | 3.02E-06 | Cathepsin Z | 15.14740238 | 0.000533032 | 21.81101994 | 0.0354 | 0.4293 |
| rs148201372 | T | A | -0.5187 | 0.01546 | 0.1063 | 1.05E-06 | Cathepsin Z | 21.49758175 | 0.000581864 | 23.81036028 | 0.0589 | 0.0854299 |
| rs148370779 | T | C | -1.7632 | 0.01316 | 0.1105 | 2.57E-57 | Cathepsin Z | 22.3469688 | 0.006187166 | 254.6118417 | 0.0717 | 0.0250098 |
| rs298724 | C | T | 0.2482 | 0.05507 | 0.0538 | 3.98E-06 | Cathepsin Z | 10.88024364 | 0.000520142 | 21.28330178 | 0.0247 | 0.5262 |
| rs36128387 | T | C | 0.439 | 0.02261 | 0.0914 | 1.55E-06 | Cathepsin Z | 18.48428008 | 0.000563768 | 23.06941858 | 0.0458 | 0.1755 |
| rs4761709 | G | A | 0.1218 | 0.40882 | 0.0249 | 1.00E-06 | Cathepsin Z | 5.035651794 | 0.000584723 | 23.92742053 | 0.0112 | 0.8293 |
| rs67845377 | T | C | 0.1675 | 0.13855 | 0.0362 | 3.72E-06 | Cathepsin Z | 7.320907427 | 0.000523231 | 21.40979366 | 0.0165 | 0.6348 |
| rs7656806 | C | A | -0.1276 | 0.48408 | 0.0245 | 1.86E-07 | Cathepsin Z | 4.954757789 | 0.000662811 | 27.1249646 | 0.0112 | 0.5739 |
| rs770140 | G | A | -0.1241 | 0.35805 | 0.0265 | 2.82E-06 | Cathepsin Z | 5.359227813 | 0.000535954 | 21.93066572 | 0.012 | 0.4018 |
| rs10016148 | A | T | -0.0896 | 0.9016 | 0.0193 | 3.39E-06 | Cathepsin L1 | 2.672614495 | 0.001122796 | 21.55268598 | 0.0182 | 0.8676 |
| rs1031153 | T | C | 0.1061 | 0.2635 | 0.0128 | 1.25E-16 | Cathepsin L1 | 1.607967363 | 0.004335552 | 68.70855713 | 0.0125 | 6.33E-07 |
| rs10469365 | A | G | 0.096 | 0.8978 | 0.0196 | 9.10E-07 | Cathepsin L1 | 2.387904286 | 0.001613859 | 23.99000416 | 0.0193 | 0.1192 |
| rs113096165 | T | C | -0.0684 | 0.208 | 0.014 | 1.10E-06 | Cathepsin L1 | 1.841838212 | 0.001377402 | 23.87020408 | 0.0136 | 0.5532 |
| rs11586939 | T | G | -0.2745 | 0.0235 | 0.0505 | 5.60E-08 | Cathepsin L1 | 5.903534238 | 0.002157675 | 29.54622096 | 0.0458 | 0.2588 |
| rs12620053 | A | C | 0.0578 | 0.5575 | 0.0122 | 2.31E-06 | Cathepsin L1 | 1.486298516 | 0.001510239 | 22.44584789 | 0.0118 | 0.8916 |
| rs143645865 | T | C | -0.1494 | 0.0346 | 0.0324 | 4.07E-06 | Cathepsin L1 | 4.375439763 | 0.00116466 | 21.26234568 | 0.0335 | 0.4075 |
| rs150011041 | T | G | -0.1849 | 0.9729 | 0.04 | 3.71E-06 | Cathepsin L1 | 5.407846152 | 0.001167791 | 21.36750625 | 0.0593 | 0.3056 |
| rs150370599 | T | C | 0.1566 | 0.0745 | 0.0211 | 1.16E-13 | Cathepsin L1 | 2.921873878 | 0.002864574 | 55.08312931 | 0.0254 | 0.1218 |
| rs1580289 | A | G | 0.048 | 0.4671 | 0.0103 | 3.50E-06 | Cathepsin L1 | 1.42628039 | 0.001131426 | 21.71740975 | 0.011 | 0.00410497 |
| rs17151689 | A | C | -0.1197 | 0.0442 | 0.0256 | 2.87E-06 | Cathepsin L1 | 3.545022336 | 0.00113894 | 21.8629303 | 0.0265 | 0.0166702 |
| rs184593554 | C | G | 0.2672 | 0.0122 | 0.0585 | 4.88E-06 | Cathepsin L1 | 7.497819733 | 0.001268541 | 20.86225144 | 0.0592 | 0.223 |
| rs2274611 | T | C | -0.1179 | 0.4415 | 0.0104 | 4.85E-30 | Cathepsin L1 | 1.440127772 | 0.006658394 | 128.5171043 | 0.0112 | 0.8739 |
| rs2921189 | A | G | 0.0534 | 0.3692 | 0.0106 | 4.08E-07 | Cathepsin L1 | 1.467822537 | 0.001321923 | 25.37878248 | 0.0113 | 0.4599 |
| rs3001922 | A | G | 0.0821 | 0.1022 | 0.0178 | 4.01E-06 | Cathepsin L1 | 2.464898343 | 0.001108286 | 21.27386062 | 0.0199 | 0.5401 |
| rs3129757 | A | C | 0.107 | 0.1514 | 0.0146 | 2.66E-13 | Cathepsin L1 | 2.021401504 | 0.002794425 | 53.71082755 | 0.0167 | 0.4458 |
| rs35049778 | A | G | 0.0711 | 0.2771 | 0.0118 | 1.67E-09 | Cathepsin L1 | 1.633991126 | 0.001890007 | 36.30573111 | 0.0121 | 0.9828 |
| rs6575449 | T | C | -0.2042 | 0.1826 | 0.0145 | 7.09E-45 | Cathepsin L1 | 1.821525528 | 0.012412848 | 198.3240904 | 0.0145 | 0.4686 |
| rs73033060 | T | G | 0.1463 | 0.0453 | 0.0307 | 1.84E-06 | Cathepsin L1 | 3.740111841 | 0.001527967 | 22.70972636 | 0.0326 | 0.894 |
| rs7412 | T | C | 0.1028 | 0.0743 | 0.0197 | 1.78E-07 | Cathepsin L1 | 2.728147727 | 0.001418011 | 27.2303847 | 0.0225 | 0.001738 |
| rs76438938 | T | C | 0.1518 | 0.0306 | 0.0307 | 7.53E-07 | Cathepsin L1 | 4.251478945 | 0.001273372 | 24.44932042 | 0.0345 | 0.6158 |
| rs76453951 | T | C | 0.0844 | 0.117 | 0.0161 | 1.65E-07 | Cathepsin L1 | 2.229544835 | 0.001431119 | 27.48103854 | 0.0186 | 0.6077 |
| rs76904798 | T | C | 0.1095 | 0.13 | 0.0154 | 1.01E-12 | Cathepsin L1 | 2.132663705 | 0.002629573 | 50.55764041 | 0.0159 | 0.0131401 |
| rs8176396 | T | C | -0.1971 | 0.0316 | 0.035 | 1.85E-08 | Cathepsin L1 | 4.396925062 | 0.002005664 | 31.71298776 | 0.0372 | 0.9304 |
| rs887945 | A | G | -0.1668 | 0.7645 | 0.0121 | 3.80E-43 | Cathepsin L1 | 1.675533273 | 0.009814045 | 190.0296428 | 0.0132 | 0.3482 |
| rs9477379 | T | C | 0.051 | 0.587 | 0.0108 | 2.29E-06 | Cathepsin L1 | 1.495517302 | 0.001161711 | 22.29938272 | 0.0111 | 0.2544 |
| rs9497486 | T | G | 0.1398 | 0.0731 | 0.0212 | 4.54E-11 | Cathepsin L1 | 2.935721622 | 0.002262799 | 43.48531506 | 0.0228 | 0.0711492 |
| rs9497576 | T | G | 0.1346 | 0.0749 | 0.0223 | 1.69E-09 | Cathepsin L1 | 2.716850285 | 0.002448795 | 36.43178025 | 0.0209 | 0.2994 |
| rs9901673 | A | C | -0.0701 | 0.1634 | 0.0139 | 4.46E-07 | Cathepsin L1 | 1.924836346 | 0.001324701 | 25.43351793 | 0.0151 | 0.2454 |

| **Table S3.** SNPs information of ten cathepsins with Parkinson's disease | | | | | | | | | | | | |
| --- | --- | --- | --- | --- | --- | --- | --- | --- | --- | --- | --- | --- |
| **Ten cathepsins** | | | | | | | | | |  | **Parkinson's disease** | |
| SNP | effect_allele | other_allele | beta | eaf | se | pval | exposure | sd | R2 | F | se | pval |
| rs113646963 | T | C | 0.1996 | 0.09708 | 0.0427 | 2.95E-06 | Cathepsin B | 8.635435004 | 0.000534001 | 21.85069846 | 0.0403 | 0.949 |
| rs11563944 | G | A | 0.1291 | 0.26799 | 0.0278 | 3.55E-06 | Cathepsin B | 5.622133328 | 0.000527039 | 21.56566689 | 0.0213 | 0.8848 |
| rs117345475 | G | A | -0.4332 | 0.02062 | 0.0933 | 3.47E-06 | Cathepsin B | 18.8685266 | 0.000526858 | 21.55825519 | 0.1069 | 0.6136 |
| rs117486267 | C | T | -0.4003 | 0.02486 | 0.0834 | 1.58E-06 | Cathepsin B | 16.86639998 | 0.000562993 | 23.0376979 | 0.0869 | 0.167 |
| rs13152767 | A | G | 0.4158 | 0.02617 | 0.0867 | 1.62E-06 | Cathepsin B | 17.53377552 | 0.000562076 | 23.00015565 | 0.151 | 0.8922 |
| rs143557119 | A | G | 0.4327 | 0.02199 | 0.0946 | 4.79E-06 | Cathepsin B | 19.13143212 | 0.000511303 | 20.92143732 | 0.0936 | 0.5272 |
| rs148930853 | C | T | 0.5516 | 0.01211 | 0.1181 | 3.02E-06 | Cathepsin B | 23.88395489 | 0.000533121 | 21.81467363 | 0.1504 | 0.0832799 |
| rs150370599 | T | C | 0.2183 | 0.08341 | 0.0457 | 1.78E-06 | Cathepsin B | 9.24214004 | 0.000557624 | 22.81786841 | 0.0325 | 0.6331 |
| rs1692819 | A | G | 0.4246 | 0.29182 | 0.0274 | 5.25E-54 | Cathepsin B | 5.541239323 | 0.005837472 | 240.1368746 | 0.0213 | 0.0001862 |
| rs36021960 | A | G | 0.1747 | 0.12637 | 0.0374 | 3.02E-06 | Cathepsin B | 7.563589442 | 0.000533236 | 21.81939003 | 0.0334 | 0.6019 |
| rs7249773 | A | G | 0.1749 | 0.15121 | 0.0351 | 6.03E-07 | Cathepsin B | 7.098448914 | 0.000606751 | 24.82935203 | 0.0235 | 0.5029 |
| rs72863882 | A | G | -0.2569 | 0.08353 | 0.0475 | 6.46E-08 | Cathepsin B | 9.606163061 | 0.000714725 | 29.25101828 | 0.0443 | 0.4063 |
| rs75773690 | A | G | -0.3945 | 0.02192 | 0.0857 | 4.17E-06 | Cathepsin B | 17.33154051 | 0.000517864 | 21.19006902 | 0.0629 | 0.0592202 |
| rs76089522 | A | G | -0.1971 | 0.10475 | 0.0421 | 2.88E-06 | Cathepsin B | 8.514093997 | 0.000535655 | 21.91841053 | 0.039 | 0.8899 |
| rs9905973 | A | G | -0.1405 | 0.271 | 0.0276 | 3.55E-07 | Cathepsin B | 5.581686326 | 0.000633239 | 25.91400179 | 0.0219 | 0.7968 |
| rs10401027 | A | G | -0.1225 | 0.45246 | 0.0259 | 2.24E-06 | Cathepsin E | 5.237886806 | 0.000546693 | 22.37034332 | 0.0222 | 0.7824 |
| rs10900907 | A | G | 0.1188 | 0.41707 | 0.0255 | 3.24E-06 | Cathepsin E | 5.156992801 | 0.000530433 | 21.70463668 | 0.0228 | 0.5776 |
| rs112918835 | T | C | 0.7062 | 0.00706 | 0.1526 | 3.72E-06 | Cathepsin E | 30.8610628 | 0.000523393 | 21.41639455 | 0.1489 | 0.7 |
| rs13089837 | C | T | -0.1152 | 0.49848 | 0.025 | 4.17E-06 | Cathepsin E | 5.055875295 | 0.000518929 | 21.233664 | 0.0225 | 0.9899 |
| rs149553686 | C | T | 0.2425 | 0.06772 | 0.0525 | 3.89E-06 | Cathepsin E | 10.61733812 | 0.000521419 | 21.33560091 | 0.0538 | 0.1314 |
| rs189009983 | T | C | 0.7055 | 0.00831 | 0.1541 | 4.68E-06 | Cathepsin E | 31.16441532 | 0.000512242 | 20.9598784 | 0.22 | 0.1089 |
| rs1936839 | C | G | 0.1562 | 0.16937 | 0.0329 | 2.00E-06 | Cathepsin E | 6.653531888 | 0.000550858 | 22.54084866 | 0.034 | 0.538801 |
| rs57135345 | T | C | 0.2535 | 0.05697 | 0.0542 | 2.95E-06 | Cathepsin E | 10.96113764 | 0.000534606 | 21.87546806 | 0.0458 | 0.7543 |
| rs57689619 | G | A | -0.2078 | 0.08618 | 0.0444 | 2.82E-06 | Cathepsin E | 8.979234524 | 0.000535305 | 21.90408652 | 0.0398 | 0.1372 |
| rs74677283 | T | C | 0.4379 | 0.02151 | 0.0882 | 6.76E-07 | Cathepsin E | 17.83712804 | 0.000602365 | 24.64976142 | 0.0754 | 0.3637 |
| rs8066936 | A | G | -0.1492 | 0.1792 | 0.0325 | 4.27E-06 | Cathepsin E | 6.572637884 | 0.000515058 | 21.07516213 | 0.0304 | 0.1774 |
| rs10745925 | C | T | -0.2874 | 0.29402 | 0.0268 | 9.12E-27 | Cathepsin F | 5.419898316 | 0.002804097 | 115.0016151 | 0.0217 | 0.0470002 |
| rs112526544 | G | A | 0.3801 | 0.02831 | 0.0766 | 6.92E-07 | Cathepsin F | 15.4912019 | 0.000601707 | 24.62284323 | 0.0661 | 0.4018 |
| rs115901379 | G | T | 0.4306 | 0.02042 | 0.0927 | 3.39E-06 | Cathepsin F | 18.74718559 | 0.000527312 | 21.57687684 | 0.0861 | 0.6256 |
| rs1260326 | C | T | -0.1642 | 0.40151 | 0.0252 | 6.92E-11 | Cathepsin F | 5.096322298 | 0.001037058 | 42.45660116 | 0.0172 | 8.24E-05 |
| rs143015877 | T | C | -0.1325 | 0.25734 | 0.0287 | 3.98E-06 | Cathepsin F | 5.804144839 | 0.000520895 | 21.31414731 | 0.0266 | 0.1069 |
| rs1791679 | A | C | 0.2349 | 0.28947 | 0.0269 | 2.51E-18 | Cathepsin F | 5.440121818 | 0.001861063 | 76.25379693 | 0.0184 | 0.1653 |
| rs183683891 | T | C | -0.5242 | 0.01536 | 0.1123 | 3.02E-06 | Cathepsin F | 22.71099183 | 0.00053249 | 21.78886062 | 0.1655 | 0.9217 |
| rs61866943 | G | T | 0.4969 | 0.01874 | 0.1012 | 9.12E-07 | Cathepsin F | 20.46618319 | 0.000589155 | 24.10887629 | 0.1103 | 0.8429 |
| rs647400 | A | G | 0.1492 | 0.19253 | 0.0314 | 2.04E-06 | Cathepsin F | 6.350179371 | 0.000551756 | 22.57762992 | 0.023 | 0.0327002 |
| rs7564167 | G | A | -0.2649 | 0.06406 | 0.0526 | 4.90E-07 | Cathepsin F | 10.63756162 | 0.000619772 | 25.36252151 | 0.0389 | 0.6067 |
| rs9796775 | G | A | -0.1172 | 0.49498 | 0.0249 | 2.45E-06 | Cathepsin F | 5.035651794 | 0.000541414 | 22.15422332 | 0.0223 | 0.8823 |
| rs10170044 | G | A | 0.115 | 0.39112 | 0.0251 | 4.79E-06 | Cathepsin G | 5.076098796 | 0.00051302 | 20.99173029 | 0.0227 | 0.03384 |
| rs114418234 | C | A | 0.3077 | 0.03719 | 0.065 | 2.24E-06 | Cathepsin G | 13.14527577 | 0.000547645 | 22.40929941 | 0.0649 | 0.1663 |
| rs116142041 | A | G | -0.3356 | 0.03645 | 0.0665 | 4.57E-07 | Cathepsin G | 13.44862829 | 0.000622356 | 25.46833852 | 0.0654 | 0.2976 |
| rs117133380 | A | C | 0.5002 | 0.01556 | 0.1065 | 2.63E-06 | Cathepsin G | 21.53802876 | 0.000539092 | 22.05911878 | 0.1597 | 0.00537898 |
| rs147099093 | A | G | 0.4271 | 0.02053 | 0.0885 | 1.38E-06 | Cathepsin G | 17.89779854 | 0.000569159 | 23.29016694 | 0.0852 | 0.4691 |
| rs192289769 | G | C | -0.5468 | 0.01364 | 0.1093 | 5.62E-07 | Cathepsin G | 22.10428679 | 0.000611589 | 25.02745493 | 0.0913 | 0.2562 |
| rs35241999 | G | A | 0.4059 | 0.02374 | 0.0869 | 2.95E-06 | Cathepsin G | 17.57422253 | 0.000533182 | 21.81717673 | 0.0907 | 0.343 |
| rs497459 | C | T | -0.1752 | 0.13466 | 0.0368 | 1.95E-06 | Cathepsin G | 7.442248434 | 0.000553912 | 22.66587902 | 0.0274 | 0.4042 |
| rs56284011 | C | G | -0.3136 | 0.03943 | 0.0656 | 1.74E-06 | Cathepsin G | 13.26661677 | 0.000558484 | 22.85306365 | 0.0506 | 0.5782 |
| rs62493038 | T | C | -0.3391 | 0.03503 | 0.0725 | 2.88E-06 | Cathepsin G | 14.66203836 | 0.000534633 | 21.87658692 | 0.0715 | 0.1121 |
| rs72773561 | C | T | -0.2529 | 0.05703 | 0.0528 | 1.70E-06 | Cathepsin G | 10.67800862 | 0.000560654 | 22.94192278 | 0.05 | 0.8288 |
| rs77893942 | A | T | -0.376 | 0.03128 | 0.0764 | 8.71E-07 | Cathepsin G | 15.4507549 | 0.000591889 | 24.22082728 | 0.0739 | 0.3463 |
| rs12911554 | T | C | -0.1198 | 0.44973 | 0.0251 | 1.82E-06 | Cathepsin H | 5.076098796 | 0.000556715 | 22.78065428 | 0.0187 | 0.2065 |
| rs146037740 | A | G | 0.4736 | 0.02292 | 0.0929 | 3.47E-07 | Cathepsin H | 18.7876326 | 0.000635074 | 25.98914304 | 0.0955 | 0.634 |
| rs147991203 | T | C | 0.3758 | 0.02671 | 0.0773 | 1.17E-06 | Cathepsin H | 15.63276641 | 0.00057758 | 23.63494324 | 0.0839 | 0.8504 |
| rs34593439 | A | G | -1.147 | 0.11066 | 0.0346 | 2.69E-241 | Cathepsin H | 6.997331408 | 0.026167806 | 1098.941662 | 0.0322 | 0.4926 |
| rs35628511 | T | C | -0.1253 | 0.3116 | 0.0272 | 4.17E-06 | Cathepsin H | 5.500792321 | 0.000518618 | 21.22092615 | 0.0195 | 0.0916305 |
| rs508807 | C | G | -0.1511 | 0.18975 | 0.032 | 2.40E-06 | Cathepsin H | 6.471520378 | 0.00054488 | 22.29610352 | 0.0279 | 0.5165 |
| rs60018174 | T | C | 0.1956 | 0.1159 | 0.0395 | 7.24E-07 | Cathepsin H | 7.988282966 | 0.000599228 | 24.52130107 | 0.0379 | 0.679301 |
| rs62013235 | A | G | 0.402 | 0.091 | 0.0432 | 1.35E-20 | Cathepsin H | 8.73655251 | 0.002112879 | 86.5933642 | 0.0332 | 0.600999 |
| rs62474230 | C | G | -0.2377 | 0.06195 | 0.0506 | 2.69E-06 | Cathepsin H | 10.2330916 | 0.000539301 | 22.06771313 | 0.0472 | 0.1387 |
| rs74342103 | T | A | 0.3766 | 0.02583 | 0.0812 | 3.47E-06 | Cathepsin H | 16.42148296 | 0.000525689 | 21.51040428 | 0.059 | 0.4907 |
| rs77977134 | C | T | -0.2721 | 0.0493 | 0.0583 | 3.02E-06 | Cathepsin H | 11.79030119 | 0.00053235 | 21.78311449 | 0.052 | 0.2141 |
| rs10902420 | G | A | -0.2097 | 0.10091 | 0.0432 | 1.23E-06 | Cathepsin O | 8.73655251 | 0.000575821 | 23.56293403 | 0.0391 | 0.708101 |
| rs146963690 | G | T | 0.6246 | 0.0101 | 0.1322 | 2.29E-06 | Cathepsin O | 26.73546856 | 0.000545522 | 22.3224084 | 0.1353 | 0.3118 |
| rs149159018 | G | A | 0.7034 | 0.01105 | 0.134 | 1.55E-07 | Cathepsin O | 27.09949158 | 0.000673304 | 27.55466474 | 0.1562 | 0.4334 |
| rs17288007 | G | A | -0.2456 | 0.06381 | 0.0521 | 2.45E-06 | Cathepsin O | 10.53644412 | 0.000543068 | 22.22190458 | 0.0513 | 0.2987 |
| rs1870736 | G | C | 0.1222 | 0.42746 | 0.0249 | 9.33E-07 | Cathepsin O | 5.035651794 | 0.000588568 | 24.08483734 | 0.0196 | 0.3185 |
| rs2439803 | G | A | 0.1763 | 0.15304 | 0.0351 | 5.13E-07 | Cathepsin O | 7.098448914 | 0.000616497 | 25.2284397 | 0.0279 | 0.9261 |
| rs4297371 | C | A | 0.1453 | 0.20694 | 0.0313 | 3.55E-06 | Cathepsin O | 6.32995587 | 0.00052665 | 21.54976574 | 0.0283 | 0.4009 |
| rs4843804 | A | G | -0.3124 | 0.0336 | 0.0673 | 3.47E-06 | Cathepsin O | 13.61041629 | 0.000526589 | 21.54725354 | 0.061 | 0.8403 |
| rs78943701 | A | G | -0.479 | 0.01536 | 0.1025 | 2.95E-06 | Cathepsin O | 20.72908871 | 0.000533703 | 21.83852469 | 0.0968 | 0.6974 |
| rs9932172 | T | C | 0.1915 | 0.10769 | 0.0401 | 1.74E-06 | Cathepsin O | 8.109623973 | 0.000557334 | 22.80598379 | 0.0365 | 0.3587 |
| rs1022239 | T | A | 0.1157 | 0.41113 | 0.025 | 3.72E-06 | Cathepsin S | 5.055875295 | 0.000523441 | 21.418384 | 0.0227 | 0.9912 |
| rs10516855 | C | T | -0.1969 | 0.09552 | 0.0421 | 2.95E-06 | Cathepsin S | 8.514093997 | 0.000534569 | 21.87395129 | 0.0332 | 0.5583 |
| rs1060435 | G | A | -0.1291 | 0.39793 | 0.0251 | 2.75E-07 | Cathepsin S | 5.076098796 | 0.000646447 | 26.45483405 | 0.0175 | 0.561699 |
| rs113108135 | C | G | 0.2349 | 0.06933 | 0.0491 | 1.70E-06 | Cathepsin S | 9.92973908 | 0.000559331 | 22.88774727 | 0.0473 | 0.666 |
| rs116623438 | C | T | -0.43 | 0.02023 | 0.0876 | 9.12E-07 | Cathepsin S | 17.71578703 | 0.000588819 | 24.0950981 | 0.0947 | 0.9857 |
| rs118010753 | C | T | 0.5018 | 0.01437 | 0.1081 | 3.47E-06 | Cathepsin S | 21.86160478 | 0.000526611 | 21.54814682 | 0.1383 | 0.2354 |
| rs12804405 | A | G | 0.612 | 0.01019 | 0.129 | 2.09E-06 | Cathepsin S | 26.08831652 | 0.000550038 | 22.50730124 | 0.1425 | 0.7558 |
| rs13150189 | A | G | 0.1556 | 0.21113 | 0.0306 | 3.47E-07 | Cathepsin S | 6.188391361 | 0.000631845 | 25.85689265 | 0.0275 | 0.6131 |
| rs13196989 | T | C | -0.1758 | 0.13099 | 0.0374 | 2.57E-06 | Cathepsin S | 7.563589442 | 0.000539969 | 22.09502702 | 0.0345 | 0.664599 |
| rs13212873 | C | T | 0.5422 | 0.0116 | 0.117 | 3.55E-06 | Cathepsin S | 23.66149638 | 0.000524841 | 21.47569874 | 0.0915 | 0.276 |
| rs13411643 | C | T | 0.1831 | 0.13712 | 0.0364 | 5.01E-07 | Cathepsin S | 7.36135443 | 0.000618321 | 25.30311104 | 0.0319 | 0.5945 |
| rs2470994 | C | T | 0.1304 | 0.28088 | 0.0281 | 3.39E-06 | Cathepsin S | 5.682803832 | 0.000526287 | 21.53488431 | 0.0244 | 0.4946 |
| rs41271951 | G | A | -0.8605 | 0.08349 | 0.0419 | 7.08E-94 | Cathepsin S | 8.473646995 | 0.010207663 | 421.7680749 | 0.0317 | 0.3351 |
| rs4313886 | C | T | 0.1687 | 0.13446 | 0.0365 | 3.89E-06 | Cathepsin S | 7.381577931 | 0.000522067 | 21.36212423 | 0.0332 | 0.8286 |
| rs4581957 | A | G | -0.1775 | 0.13414 | 0.0365 | 1.15E-06 | Cathepsin S | 7.381577931 | 0.000577921 | 23.64890223 | 0.0341 | 0.8421 |
| rs6657328 | C | G | -0.1291 | 0.2816 | 0.0283 | 4.90E-06 | Cathepsin S | 5.723250834 | 0.000508589 | 20.81036097 | 0.0251 | 0.1917 |
| rs73099998 | T | C | -0.146 | 0.20811 | 0.0308 | 2.19E-06 | Cathepsin S | 6.228838364 | 0.000549129 | 22.47006241 | 0.0282 | 0.03724 |
| rs74804137 | C | T | -0.5178 | 0.01757 | 0.0987 | 1.58E-07 | Cathepsin S | 19.96059567 | 0.000672521 | 27.52262082 | 0.1079 | 0.1663 |
| rs7614425 | A | G | -0.2082 | 0.09254 | 0.0436 | 1.82E-06 | Cathepsin S | 8.817446515 | 0.000557256 | 22.80281542 | 0.0398 | 0.2442 |
| rs77792819 | G | A | 0.3147 | 0.04096 | 0.0661 | 1.95E-06 | Cathepsin S | 13.36773428 | 0.000553935 | 22.66681849 | 0.0609 | 0.616601 |
| rs78767885 | C | T | 0.2238 | 0.08725 | 0.0477 | 2.69E-06 | Cathepsin S | 9.646610063 | 0.00053797 | 22.0132115 | 0.0337 | 0.5101 |
| rs78767885 | C | T | 0.2238 | 0.08725 | 0.0477 | 2.69E-06 | Cathepsin S | 9.646610063 | 0.00053797 | 22.0132115 | 0.0337 | 0.5101 |
| rs989576 | T | C | 0.1307 | 0.29843 | 0.0278 | 2.57E-06 | Cathepsin S | 5.622133328 | 0.000540176 | 22.10352725 | 0.0251 | 0.7583 |
| rs10817163 | A | T | 0.1983 | 0.22045 | 0.0304 | 7.08E-11 | Cathepsin L2 | 6.147944359 | 0.001039334 | 42.54987232 | 0.0268 | 0.0926403 |
| rs114113108 | C | G | -0.5922 | 0.0129 | 0.1172 | 4.37E-07 | Cathepsin L2 | 23.70194338 | 0.000623906 | 25.53180876 | 0.1165 | 0.9903 |
| rs116407656 | C | T | 0.4014 | 0.02439 | 0.0856 | 2.75E-06 | Cathepsin L2 | 17.31131701 | 0.000537381 | 21.98908747 | 0.0912 | 0.7471 |
| rs116407656 | C | T | 0.4014 | 0.02439 | 0.0856 | 2.75E-06 | Cathepsin L2 | 17.31131701 | 0.000537381 | 21.98908747 | 0.0912 | 0.7471 |
| rs117714361 | G | A | -0.3452 | 0.02685 | 0.0756 | 4.90E-06 | Cathepsin L2 | 15.28896689 | 0.000509548 | 20.84961227 | 0.0642 | 0.6505 |
| rs13068566 | G | A | 0.1157 | 0.46617 | 0.0247 | 2.88E-06 | Cathepsin L2 | 4.995204792 | 0.000536227 | 21.94182825 | 0.0223 | 0.796 |
| rs148608463 | A | G | -0.1178 | 0.35415 | 0.0257 | 4.57E-06 | Cathepsin L2 | 5.197439803 | 0.000513464 | 21.00991688 | 0.0176 | 0.4567 |
| rs151179824 | A | G | 0.4449 | 0.02158 | 0.0943 | 2.34E-06 | Cathepsin L2 | 19.07076161 | 0.000543968 | 22.25878354 | 0.1035 | 0.9158 |
| rs1523319 | G | C | -0.1292 | 0.28541 | 0.0279 | 3.55E-06 | Cathepsin L2 | 5.642356829 | 0.00052408 | 21.44453437 | 0.0224 | 0.088481 |
| rs2302837 | G | A | -0.2541 | 0.07506 | 0.0498 | 3.31E-07 | Cathepsin L2 | 10.07130359 | 0.000636184 | 26.03458412 | 0.0446 | 0.565999 |
| rs7669728 | C | T | -0.1301 | 0.43633 | 0.0254 | 3.09E-07 | Cathepsin L2 | 5.1367693 | 0.000641087 | 26.23536797 | 0.0225 | 0.0477101 |
| rs7898416 | G | A | -0.2277 | 0.0705 | 0.0489 | 3.24E-06 | Cathepsin L2 | 9.889292077 | 0.000529891 | 21.68244947 | 0.0439 | 0.1262 |
| rs10745925 | C | T | -0.3624 | 0.29402 | 0.0266 | 2.29E-42 | Cathepsin Z | 5.379451314 | 0.004518092 | 185.615015 | 0.0217 | 0.0470002 |
| rs10761760 | C | A | 0.1321 | 0.44944 | 0.0258 | 3.09E-07 | Cathepsin Z | 5.217663305 | 0.000640614 | 26.21598762 | 0.0168 | 0.528901 |
| rs1135945 | A | G | 0.1385 | 0.20839 | 0.0301 | 4.17E-06 | Cathepsin Z | 6.087273855 | 0.000517428 | 21.17222768 | 0.0232 | 0.726099 |
| rs114675081 | G | A | 0.5153 | 0.01467 | 0.1075 | 1.66E-06 | Cathepsin Z | 21.74026377 | 0.000561524 | 22.97753077 | 0.103 | 0.6207 |
| rs116920068 | A | G | -0.3498 | 0.0289 | 0.0749 | 3.02E-06 | Cathepsin Z | 15.14740238 | 0.000533032 | 21.81101994 | 0.0678 | 0.9453 |
| rs148201372 | T | A | -0.5187 | 0.01546 | 0.1063 | 1.05E-06 | Cathepsin Z | 21.49758175 | 0.000581864 | 23.81036028 | 0.1589 | 0.9492 |
| rs148370779 | T | C | -1.7632 | 0.01316 | 0.1105 | 2.57E-57 | Cathepsin Z | 22.3469688 | 0.006187166 | 254.6118417 | 0.1238 | 0.9094 |
| rs298724 | C | T | 0.2482 | 0.05507 | 0.0538 | 3.98E-06 | Cathepsin Z | 10.88024364 | 0.000520142 | 21.28330178 | 0.046 | 0.541101 |
| rs36128387 | T | C | 0.439 | 0.02261 | 0.0914 | 1.55E-06 | Cathepsin Z | 18.48428008 | 0.000563768 | 23.06941858 | 0.094 | 0.836 |
| rs4761709 | G | A | 0.1218 | 0.40882 | 0.0249 | 1.00E-06 | Cathepsin Z | 5.035651794 | 0.000584723 | 23.92742053 | 0.0184 | 0.668499 |
| rs67845377 | T | C | 0.1675 | 0.13855 | 0.0362 | 3.72E-06 | Cathepsin Z | 7.320907427 | 0.000523231 | 21.40979366 | 0.0285 | 0.459 |
| rs7656806 | C | A | -0.1276 | 0.48408 | 0.0245 | 1.86E-07 | Cathepsin Z | 4.954757789 | 0.000662811 | 27.1249646 | 0.0223 | 0.3426 |
| rs770140 | G | A | -0.1241 | 0.35805 | 0.0265 | 2.82E-06 | Cathepsin Z | 5.359227813 | 0.000535954 | 21.93066572 | 0.0272 | 0.5346 |
| rs10016148 | A | T | -0.0896 | 0.9016 | 0.0193 | 3.39E-06 | Cathepsin L1 | 2.672614495 | 0.001122796 | 21.55268598 | 0.0371 | 0.1307 |
| rs1031153 | T | C | 0.1061 | 0.2635 | 0.0128 | 1.25E-16 | Cathepsin L1 | 1.607967363 | 0.004335552 | 68.70855713 | 0.0191 | 0.0181201 |
| rs10469365 | A | G | 0.096 | 0.8978 | 0.0196 | 9.10E-07 | Cathepsin L1 | 2.387904286 | 0.001613859 | 23.99000416 | 0.0312 | 0.002071 |
| rs113096165 | T | C | -0.0684 | 0.208 | 0.014 | 1.10E-06 | Cathepsin L1 | 1.841838212 | 0.001377402 | 23.87020408 | 0.0225 | 0.865 |
| rs11586939 | T | G | -0.2745 | 0.0235 | 0.0505 | 5.60E-08 | Cathepsin L1 | 5.903534238 | 0.002157675 | 29.54622096 | 0.0849 | 0.8864 |
| rs12620053 | A | C | 0.0578 | 0.5575 | 0.0122 | 2.31E-06 | Cathepsin L1 | 1.486298516 | 0.001510239 | 22.44584789 | 0.0231 | 0.5439 |
| rs143645865 | T | C | -0.1494 | 0.0346 | 0.0324 | 4.07E-06 | Cathepsin L1 | 4.375439763 | 0.00116466 | 21.26234568 | 0.0665 | 0.1334 |
| rs150011041 | T | G | -0.1849 | 0.9729 | 0.04 | 3.71E-06 | Cathepsin L1 | 5.407846152 | 0.001167791 | 21.36750625 | 0.1494 | 0.9669 |
| rs150370599 | T | C | 0.1566 | 0.0745 | 0.0211 | 1.16E-13 | Cathepsin L1 | 2.921873878 | 0.002864574 | 55.08312931 | 0.0325 | 0.6331 |
| rs1580289 | A | G | 0.048 | 0.4671 | 0.0103 | 3.50E-06 | Cathepsin L1 | 1.42628039 | 0.001131426 | 21.71740975 | 0.0224 | 0.2895 |
| rs17151689 | A | C | -0.1197 | 0.0442 | 0.0256 | 2.87E-06 | Cathepsin L1 | 3.545022336 | 0.00113894 | 21.8629303 | 0.0414 | 0.5455 |
| rs181283433 | C | G | 0.3521 | 0.9909 | 0.0768 | 4.50E-06 | Cathepsin L1 | 8.67806017 | 0.001643767 | 21.0188819 | 0.2695 | 0.7247 |
| rs184593554 | C | G | 0.2672 | 0.0122 | 0.0585 | 4.88E-06 | Cathepsin L1 | 7.497819733 | 0.001268541 | 20.86225144 | 0.1729 | 0.1357 |
| rs2274611 | T | C | -0.1179 | 0.4415 | 0.0104 | 4.85E-30 | Cathepsin L1 | 1.440127772 | 0.006658394 | 128.5171043 | 0.0198 | 0.5444 |
| rs2921189 | A | G | 0.0534 | 0.3692 | 0.0106 | 4.08E-07 | Cathepsin L1 | 1.467822537 | 0.001321923 | 25.37878248 | 0.023 | 0.9091 |
| rs3001922 | A | G | 0.0821 | 0.1022 | 0.0178 | 4.01E-06 | Cathepsin L1 | 2.464898343 | 0.001108286 | 21.27386062 | 0.0385 | 0.0171002 |
| rs3129757 | A | C | 0.107 | 0.1514 | 0.0146 | 2.66E-13 | Cathepsin L1 | 2.021401504 | 0.002794425 | 53.71082755 | 0.0275 | 0.3527 |
| rs35049778 | A | G | 0.0711 | 0.2771 | 0.0118 | 1.67E-09 | Cathepsin L1 | 1.633991126 | 0.001890007 | 36.30573111 | 0.025 | 0.745399 |
| rs554689923 | C | G | 0.3594 | 0.9862 | 0.0755 | 1.93E-06 | Cathepsin L1 | 6.920924848 | 0.002690057 | 22.66012192 | 0.1113 | 0.6584 |
| rs6575449 | T | C | -0.2042 | 0.1826 | 0.0145 | 7.09E-45 | Cathepsin L1 | 1.821525528 | 0.012412848 | 198.3240904 | 0.0241 | 0.82 |
| rs73033060 | T | G | 0.1463 | 0.0453 | 0.0307 | 1.84E-06 | Cathepsin L1 | 3.740111841 | 0.001527967 | 22.70972636 | 0.06 | 0.3879 |
| rs7412 | T | C | 0.1028 | 0.0743 | 0.0197 | 1.78E-07 | Cathepsin L1 | 2.728147727 | 0.001418011 | 27.2303847 | 0.0313 | 0.7275 |
| rs76438938 | T | C | 0.1518 | 0.0306 | 0.0307 | 7.53E-07 | Cathepsin L1 | 4.251478945 | 0.001273372 | 24.44932042 | 0.0547 | 0.2864 |
| rs76453951 | T | C | 0.0844 | 0.117 | 0.0161 | 1.65E-07 | Cathepsin L1 | 2.229544835 | 0.001431119 | 27.48103854 | 0.0287 | 0.5553 |
| rs76904798 | T | C | 0.1095 | 0.13 | 0.0154 | 1.01E-12 | Cathepsin L1 | 2.132663705 | 0.002629573 | 50.55764041 | 0.0235 | 9.22E-09 |
| rs8176396 | T | C | -0.1971 | 0.0316 | 0.035 | 1.85E-08 | Cathepsin L1 | 4.396925062 | 0.002005664 | 31.71298776 | 0.0718 | 0.4792 |
| rs887945 | A | G | -0.1668 | 0.7645 | 0.0121 | 3.80E-43 | Cathepsin L1 | 1.675533273 | 0.009814045 | 190.0296428 | 0.0201 | 0.1016 |
| rs9477379 | T | C | 0.051 | 0.587 | 0.0108 | 2.29E-06 | Cathepsin L1 | 1.495517302 | 0.001161711 | 22.29938272 | 0.0224 | 0.677901 |
| rs9497486 | T | G | 0.1398 | 0.0731 | 0.0212 | 4.54E-11 | Cathepsin L1 | 2.935721622 | 0.002262799 | 43.48531506 | 0.0361 | 0.3025 |
| rs9497576 | T | G | 0.1346 | 0.0749 | 0.0223 | 1.69E-09 | Cathepsin L1 | 2.716850285 | 0.002448795 | 36.43178025 | 0.0327 | 0.1987 |
| rs9901673 | A | C | -0.0701 | 0.1634 | 0.0139 | 4.46E-07 | Cathepsin L1 | 1.924836346 | 0.001324701 | 25.43351793 | 0.0219 | 0.0307298 |

| Table S4. Reverse MR analysis of cathepsins and neurodegenerative diseases | | | | | | | | | | |
| --- | --- | --- | --- | --- | --- | --- | --- | --- | --- | --- |
| exposure | outcome | SNPs | Inverse variance weighted | |  | MR-Egger | |  | Weighted median | |
|  |  |  | OR (95%CI) | *p_value* |  | OR (95%CI) | *p_value* |  | OR (95%CI) | *p_value* |
| AD | Cathepsin B | 68 | 1.031（0.949-1.120） | 0.464 |  | 0.975（0.829-1.146） | 0.761 |  | 1.006（0.885-1.143） | 0.924 |
|  | Cathepsin E | 68 | 0.985（0.908-1.068） | 0.724 |  | 0.951（0.812-1.113） | 0.535 |  | 1.027(0.908-1.161) | 0.667 |
|  | Cathepsin F | 68 | 0.980(0.900-1.068) | 0.656 |  | 1.034 (0.875-1.223) | 0.689 |  | 1.018(0.890-1.164) | 0.790 |
|  | Cathepsin G | 68 | 0.979(0.903-1.061) | 0.609 |  | 0.946(0.808-1.108) | 0.497 |  | 0.982(0.868-1.111) | 0.776 |
|  | Cathepsin H | 68 | 0.977（0.901-1.060） | 0.587 |  | 0.943(0.805-1.104) | 0.471 |  | 0.941（0.830-1.067） | 0.349 |
|  | Cathepsin L2 | 68 | 1.034(0.949-1.127) | 0.435 |  | 0.992(0.839-1.174) | 0.934 |  | 1.043(0.922-1.180) | 0.498 |
|  | Cathepsin O | 68 | 0.987(0.910-1.070) | 0.755 |  | 0.975(0.832-1.142) | 0.755 |  | 1.048(0.925-1.186) | 0.459 |
|  | Cathepsin S | 68 | 0.963(0.888-1.044) | 0.364 |  | 0.885(0.756-1.036) | 0.134 |  | 0.939(0.830-1.062) | 0.322 |
|  | Cathepsin Z | 68 | 1.038(0.957-1.125) | 0.363 |  | 1.059(0.903-1.242) | 0.478 |  | 0.994(0.879-1.124) | 0.930 |
| PD | Cathepsin B | 18 | 0.951(0.876-1.031) | 0.225 |  | 0.931(0.773-1.120) | 0.462 |  | 0.927(0.834-1.032) | 0.168 |
|  | Cathepsin E | 18 | 1.061(0.964-1.167) | 0.219 |  | 1.225(0.992-1.513) | 0.077 |  | 1.050（0.925-1.192） | 0.447 |
|  | Cathepsin F | 18 | 1.000（0.909-1.101） | 0.989 |  | 1.008（0.804-1.264） | 0.939 |  | 0.944（0.838-1.064） | 0.349 |
|  | Cathepsin G | 18 | 0.972（0.880-1.074） | 0.587 |  | 1.056（0.839-1.329） | 0.648 |  | 1.013（0.893-1.150） | 0.832 |
|  | Cathepsin H | 18 | 0.947（0.861-1.041） | 0.265 |  | 0.988（0.790-1.235） | 0.918 |  | 0.901（0.793-1.024） | 0.112 |
|  | Cathepsin L2 | 18 | 0.959（0.880-1.045） | 0.347 |  | 0.869（0.715-1.057） | 0.181 |  | 1.007（0.898-1.130） | 0.895 |
|  | Cathepsin O | 18 | 0.957（0.869-1.054） | 0.381 |  | 1.024（0.818-1.283） | 0.833 |  | 0.951（0.841-1.074） | 0.422 |
|  | Cathepsin S | 18 | 0.941（0.868-1.021） | 0.145 |  | 0.940（0.781-1.132） | 0.527 |  | 0.885（0.788-0.994） | 0.039 |
|  | Cathepsin Z | 18 | 1.013（0.907-1.131） | 0.811 |  | 0.880（0.687-1.128） | 0.330 |  | 1.035（0.918-1.168） | 0.566 |
| ALS | Cathepsin B | 12 | 0.923（0.785-1.086） | 0.335 |  | 0.993（0.688-1.432） | 0.971 |  | 0.954（0.766-1.188） | 0.674 |
|  | Cathepsin E | 12 | 1.039（0.884-1.222） | 0.638 |  | 1.077（0.746-1.554） | 0.699 |  | 0.975（0.791-1.203） | 0.817 |
|  | Cathepsin F | 12 | 1.030（0.876-1.211） | 0.715 |  | 0.853（0.591-1.230） | 0.415 |  | 0.957（0.766-1.195） | 0.701 |
|  | Cathepsin G | 12 | 1.000（0.981-1.019） | 0.864 |  | 1.016（0.845-1.220） | 0.302 |  | 1.045（0.838-1.303） | 0.691 |
|  | Cathepsin H | 12 | 0.886（0.754-1.043） | 0.146 |  | 0.715（0.496-1.032） | 0.104 |  | 0.887（0.715-1.100） | 0.276 |
|  | Cathepsin L2 | 12 | 0.917（0.780-1.079） | 0.299 |  | 0.857（0.594-1.237） | 0.430 |  | 0.865（0.699-1.071） | 0.184 |
|  | Cathepsin O | 12 | 1.005（0.970-1.042） | 0.212 |  | 0.901（0.766-1.060） | 0.674 |  | 0.922（0.639-1.330） | 0.622 |
|  | Cathepsin S | 12 | 1.009（0.847-1.201） | 0.914 |  | 1.134（0.756-1.702） | 0.555 |  | 1.100（0.879-1.377） | 0.402 |
|  | Cathepsin Z | 12 | 0.897（0.732-1.101） | 0.301 |  | 1.051（0.655-1.686） | 0.838 |  | 0.843（0.675-1.053） | 0.133 |
